# Dietary, lifestyle, and host determinants of microbiota-host interactions

**DOI:** 10.3389/frmbi.2026.1975060

**Published:** 2026-10-08

**Authors:** Alma Haydee Astorga Gaxiola, Luis Martin Sánchez Magaña, Liliana León López, Cuauhtémoc Reyes Moreno, Saraid Mora Rochín

**Affiliations:** 1 Facultad de Ciencias Químico-Biológicas, Universidad Autónoma de Sinaloa, Culiacán, Sinaloa, C.P., Mexico; 2 Instituto Tecnológico de Culiacán, Tecnológico Nacional de México, Culiacán, C.P., Sinaloa, Mexico; 3 Programa de Posgrado Integral en Biotecnología, Facultad de Ciencias Químico Biológicas, Universidad Autónoma de Sinaloa, Culiacán, Sinaloa, C.P., Mexico; 4 Maestría en Ciencia y Tecnología de Alimentos, Facultad de Ciencias Químico Biológicas, Universidad Autónoma de Sinaloa, Culiacán, Sinaloa, C.P., Mexico

**Keywords:** dietary factors, gut microbiota, interindividual variability, lifestyle factors, personalized nutrition

## Abstract

The gut microbiota is a dynamic ecosystem shaped by dietary factors, lifestyle factors, environmental exposures, and host characteristics, with important implications for metabolism, immune regulation, intestinal barrier function, and host–microbiota interactions. This narrative review examines how dietary patterns, specific food components, physical activity, sleep and circadian rhythms, aging, and medication exposure modulate gut microbial composition and function, with particular emphasis on interindividual variability and personalized nutrition. A literature search was conducted in Scopus, ScienceDirect, PubMed, and Google Scholar for publications from 2000 to 2026, with emphasis on recent peer-reviewed evidence. Current evidence indicates that dietary patterns and individual food components can modify microbial community structure, metabolic pathways, and bioactive metabolite production, although responses vary substantially across individuals. Physical activity may modify microbial ecology, intestinal physiology, barrier function, and microbial metabolism, whereas sleep disruption and circadian misalignment may alter microbial rhythmicity and host metabolic regulation. Aging and antibiotic exposure are associated with context-dependent changes in microbial composition, function, resilience, and, with antibiotics, the gut resistome. Across these determinants, interindividual variability emerges as a central feature driven by host characteristics, habitual diet, lifestyle, medication history, baseline microbiota, and microbial functional capacity. Personalized nutrition therefore represents a promising but evolving framework in which microbiome data may be integrated with host and environmental characteristics to predict differential responses. Future research should prioritize longitudinal human studies, standardized methodologies, multi-omics integration, and prospective validation of microbiome-informed interventions to establish clinically meaningful benefits.

## 1 Introduction

The gut microbiota is a dynamic and complex microbial ecosystem that plays an important role in host metabolism, immune regulation, intestinal barrier integrity, and communication between the gastrointestinal tract and distant organs. Its composition and function are continuously shaped by the interplay among dietary exposures, lifestyle behaviors, environmental conditions, medications, and host characteristics. Consequently, host–microbiota interactions cannot be fully understood by examining individual determinants in isolation; they require consideration of their interconnected effects within a complex biological system (David et al., 2014; Gupta et al., 2017; Ussar et al., 2016).

Among these determinants, diet represents one of the most influential factors affecting microbial ecology and function. Dietary patterns rich in plant-derived foods and complex carbohydrates provide substrates for microbial fermentation and short-chain fatty acid production. In contrast, Western dietary patterns, characterized by high intakes of saturated fat, refined sugars, sodium, and ultra-processed foods, have been associated with alterations in microbial ecology and host metabolic and inflammatory responses (Las Heras et al., 2022; Senghor et al., 2018). However, diet effects cannot be attributed to individual nutrients alone. Fiber, polyphenols, proteins, lipids, vitamins, minerals, and fermented foods may interact with microbial communities according to their chemical properties, food matrix, dose, and the metabolic capabilities of the resident microbiota. Thus, microbial responses to dietary exposure depend not only on what is consumed but also on how dietary components interact with one another and with the existing microbial ecosystem.

Beyond diet, lifestyle, environmental, and physiological factors further contribute to variation in the gut microbiota. Physical activity has been associated with changes in intestinal physiology, barrier integrity, immune function, and microbial metabolism, while sleep and circadian rhythms help organize microbial activity in time and support host metabolic homeostasis (Campaniello et al., 2022; Lotti et al., 2023). Bidirectional communication between the gut microbiota and the nervous system, commonly described as the microbiota–gut–brain axis, provides another example of the complex relationship between microbial activity and host physiology, with potential implications for mental health (Cooke et al., 2022). Similarly, aging brings heterogeneous changes in microbial composition and function, while medication exposure, particularly to antibiotics, can substantially alter microbial communities and the intestinal resistome (Ling et al., 2022; Lathakumari et al., 2024).

A key feature underlying these interactions is substantial interindividual variability in microbial and host responses to similar exposures. Factors such as age, sex, metabolic status, habitual diet, physical activity, medication history, environmental conditions, and baseline microbiota may influence both the magnitude and direction of these responses (Gupta et al., 2017; Ussar et al., 2016). Furthermore, taxonomic composition alone does not necessarily account for functional differences between individuals. Distinct microbial communities may exhibit overlapping metabolic capacities, whereas communities with similar taxonomic profiles may differ in their metabolic activity. Integrating microbiota characteristics with dietary, clinical, metabolomic, and other multi-omics information may therefore provide a more comprehensive understanding of differential responses to nutritional interventions (Kviatcovsky et al., 2021).

Although numerous reviews have examined specific dietary, lifestyle, environmental, or host-related determinants of the gut microbiota, these factors are often considered separately despite their substantial interdependence. This fragmented perspective may limit the interpretation of microbial responses that emerge from the combined influence of multiple exposures and host characteristics. An integrated perspective is therefore needed to distinguish relatively consistent associations from context-dependent responses and to understand better their implications for host health and the emerging field of personalized nutrition.

Accordingly, this narrative review synthesizes current evidence on how dietary patterns and food components, lifestyle behaviors, environmental and physiological factors, and medication exposure modulate the gut microbiota. It emphasizes the biological basis of interindividual variability and how interactions among microbial, dietary, host, and environmental factors may influence health-related outcomes. By bringing these determinants together, the review aims to provide an integrated perspective on host–microbiota interactions and highlight the challenges and opportunities of translating microbiome variability into personalized nutritional strategies.

## 2 Methods

### 2.1 Literature search and evidence synthesis

This article presents a narrative review of current evidence on how dietary, lifestyle, environmental, and host-related factors modulate the gut microbiota. The literature was compiled between April and July 2026 using Scopus, ScienceDirect, PubMed, and Google Scholar. Publications published between 2000 and 2026 were considered, with emphasis on recent evidence. Searches combined general terms related to gut microbiota, health, and lifestyle with topic-specific terms addressing dietary patterns and food components, physical activity, mental health, sleep and circadian rhythms, aging, medication exposure, interindividual variability, and personalized nutrition.

Peer-reviewed original research articles, systematic reviews, meta-analysis, and relevant narrative reviews were considered, whereas book chapters, editorials, and conference abstracts were excluded. Additional relevant studies were identified through reference-list screening and targeted searches of specific microbial taxa, metabolites, mechanisms, and host–microbiota interactions. The selected literature was critically evaluated and qualitatively synthesized according to microbial composition and function, microbial metabolites, host–microbiota interactions, associated health outcomes, and sources of interindividual variability. When findings were inconsistent, the interpretation considered differences in study populations, exposures, experimental designs, and analytical approaches.

## 3 Dietary factors: the primary modulators

Diet is one of the strongest and most dynamic determinants of gut microbiota composition and function. Modern dietary patterns characterized by high consumption of energy-dense, highly processed foods and reduced intake of fiber-rich plant foods have been associated with alterations in microbial ecology and host metabolic and inflammatory responses (Las Heras et al., 2022; Gonzalez-Olmo et al., 2021). However, dietary effects cannot be explained solely by individual macronutrient intake. Food quality, dietary pattern, processing, nutrient interactions, and the metabolic capacity of the resident microbiota collectively influence microbial and host responses. Thus, dietary modulation of the gut microbiota depends on both the characteristics of the dietary exposure and the biological context in which it occurs.

The human gastrointestinal tract harbors a diverse microbial ecosystem composed of bacteria, archaea, fungi, viruses, and other microorganisms. Rather than a fixed set of species, the gut microbiota exhibits substantial taxonomic and functional diversity across individuals and anatomical sites. This community contributes to nutrient metabolism, immune development, intestinal barrier integrity, and microbial metabolite production. Its collective genetic repertoire, commonly referred to as the gut microbiome, expands the metabolic potential available to the host (Cataldi et al., 2022; Lotti et al., 2023).

Poor dietary patterns can disrupt gut microbiota homeostasis by altering the intestinal environment, barrier integrity, and microbial metabolism (He et al., 2026; Vaughn et al., 2017; O’Hara and Shanahan, 2006). However, these effects depend on dietary pattern, host characteristics, and baseline microbial composition. The gut microbiota is highly individualized and dynamically shaped by diet, host genetics, lifestyle, medication exposure, and environmental factors (David et al., 2014; Gupta et al., 2017). Microbial metabolism also contributes to host physiology by degrading complex polysaccharides and polyphenols and synthesizing vitamins, particularly B vitamins and vitamin K (Zhai et al., 2022). Dietary interventions can produce measurable microbial changes over relatively short periods, although their magnitude, persistence, and clinical relevance vary among individuals.

Modern DNA sequencing technologies have enabled investigation of how entire dietary patterns shape gut microbiota diversity and function, including differences between vegetarian and omnivorous populations (Zimmer et al., 2012; Matijašić et al., 2014). Dietary habits vary globally according to agricultural practices, cultural heritage, and socioeconomic development, contributing to distinct dietary patterns and associated microbial profiles (Senghor et al., 2018). Despite substantial interindividual variation, gut microbial communities can be broadly characterized by configurations dominated by genera such as Bacteroides, Prevotella, or Ruminococcus (Costea et al., 2018). The Bacteroides enterotype has been associated with high-fat, high-protein Western diets, whereas Prevotella-dominant profiles are more commonly observed with diets rich in complex carbohydrates, fruits, and vegetables (Wu et al., 2011).

### 3.1 Plant-based and Mediterranean diets

Dietary habits serve as a primary determinant of long-term gut microbiota composition in humans. Transitioning to either a strictly plant-based or animal-based diet triggers rapid taxonomic shifts; notably, an animal-based diet selectively enriches bile-tolerant microbes while suppressing taxa that ferment plant-derived polysaccharides (Las Heras et al., 2022; David et al., 2014). Similarly, highly balanced dietary archetypes, such as the Mediterranean diet, are characterized by high consumption of fiber-rich fruits, vegetables, olive oil, nuts, legumes, and whole grains, together with moderate consumption of fish and seafood, poultry, dairy products, and eggs (Du et al., 2021; Senghor et al., 2018). Crucially, greater adherence to the Mediterranean diet is associated with higher concentrations of microbial short-chain fatty acids (SCFAs) (García-Mantrana et al., 2018).

The Mediterranean diet is widely considered a dietary pattern that contrasts substantially with the Western dietary pattern in terms of food quality, plant-food intake, and fat sources. Numerous studies indicate that this fiber-rich dietary pattern is associated with greater microbial diversity and with changes in the abundance of taxa involved in carbohydrate fermentation and host–microbiota interactions, including *Bacteroides* and bifidobacteria such as *Bifidobacterium breve* and *Bifidobacterium longum* (Nova et al., 2022; Kundra et al., 2021) (**Table 1**).

**Table 1:** Dietary patterns, gut microbiota features, microbial metabolites, and reported health associations.

Dietary pattern	Reported microbial changes	Key microbial metabolites/functional features	Commonly reported health and metabolic associations	Evidence and limitations	References
Mediterranean diet	↑ *Prevotella*, *Faecalibacterium prausnitzii*, *Bifidobacterium*, *Lactobacillus*, and other taxa involved in carbohydrate fermentation; changes in community diversity and composition have also been reported.	↑ Acetate, propionate, and butyrate; increased microbial metabolism of dietary polyphenols; potential modulation of bile-acid metabolism.	Associated with improved intestinal barrier function, lower inflammatory tone, and more favorable metabolic and cardiovascular risk profiles.	Human observational and intervention evidence; findings are generally consistent for selected microbial and metabolic features, but effects vary with adherence, intervention duration, baseline microbiota, host characteristics, and methodology.	(Barber et al., 2023; Gundogdu and Nalbantoglu, 2023; García-Montero et al., 2021)
Western diet	↑ Certain Proteobacteria and Enterobacteriaceae and changes in *Bacteroides*, *Alistipes*, and other taxa; ↓ or reduced abundance of some SCFA-producing and fiber-associated taxa, including *Faecalibacterium prausnitzii* and *Roseburia*, have been reported in some studies.	Alterations in SCFA production; ↑ microbial metabolites associated with animal-protein and fat metabolism, including TMAO precursors and secondary bile acids; ↑ potentially pro-inflammatory microbial products.	Associated with altered intestinal barrier function, increased metabolic endotoxemia and inflammatory signaling, and greater risk of obesity and cardiometabolic dysfunction.	Human observational and experimental evidence: microbial and metabolic alterations are context-dependent and may reflect the combined effects of fat, refined carbohydrates, ultra-processed foods, and overall dietary pattern rather than a single dietary component.	(Yang et al., 2020; Beam et al., 2021; García-Montero et al., 2021; Ramos and Martín, 2021; Nova et al., 2022)

↑, increase or enrichment; ↓, decrease or reduction. Taxonomic changes represent findings reported across studies and should not be interpreted as universal signatures of a given dietary pattern.

However, findings remain somewhat inconsistent. Although Mediterranean dietary patterns are frequently associated with favorable changes in gut microbial diversity and function, intervention studies do not consistently show substantial changes in overall community composition. Differences in intervention duration, baseline microbiota, dietary adherence, host characteristics, and methodology may contribute to this variability (Du et al., 2021).

Nevertheless, high adherence to the Mediterranean diet has been associated with reduced Escherichia coli counts and an increased Bifidobacteria-to-E. coli ratio, suggesting that dietary adherence may influence specific taxa even when broader community changes are limited (Mitsou et al., 2017).

Overall, adherence to a Mediterranean dietary pattern may support gut microbial homeostasis and represents a promising strategy for promoting metabolic and inflammatory health (Du et al., 2021).

### 3.2 Western diet

The Western dietary pattern is characterized primarily by a high consumption of energy-dense, highly processed, and ultra-processed foods (Las Heras et al., 2022; Clemente-Suárez et al., 2023). Compared with traditional rural diets, this pattern is generally characterized by high intake of animal-derived products, refined sugars, saturated fat, and sodium, alongside low intake of plant-derived dietary fiber and complex carbohydrates. These characteristics may influence gut microbial composition and function, intestinal barrier integrity, and host metabolic and inflammatory responses. However, the magnitude and direction of these effects vary according to the type and amount of dietary fat and carbohydrate, food processing, habitual dietary patterns, and individual host characteristics (**Figure 1**) (He et al., 2026; Reiss et al., 2016; Lairon, 2007).

**Figure 1:**
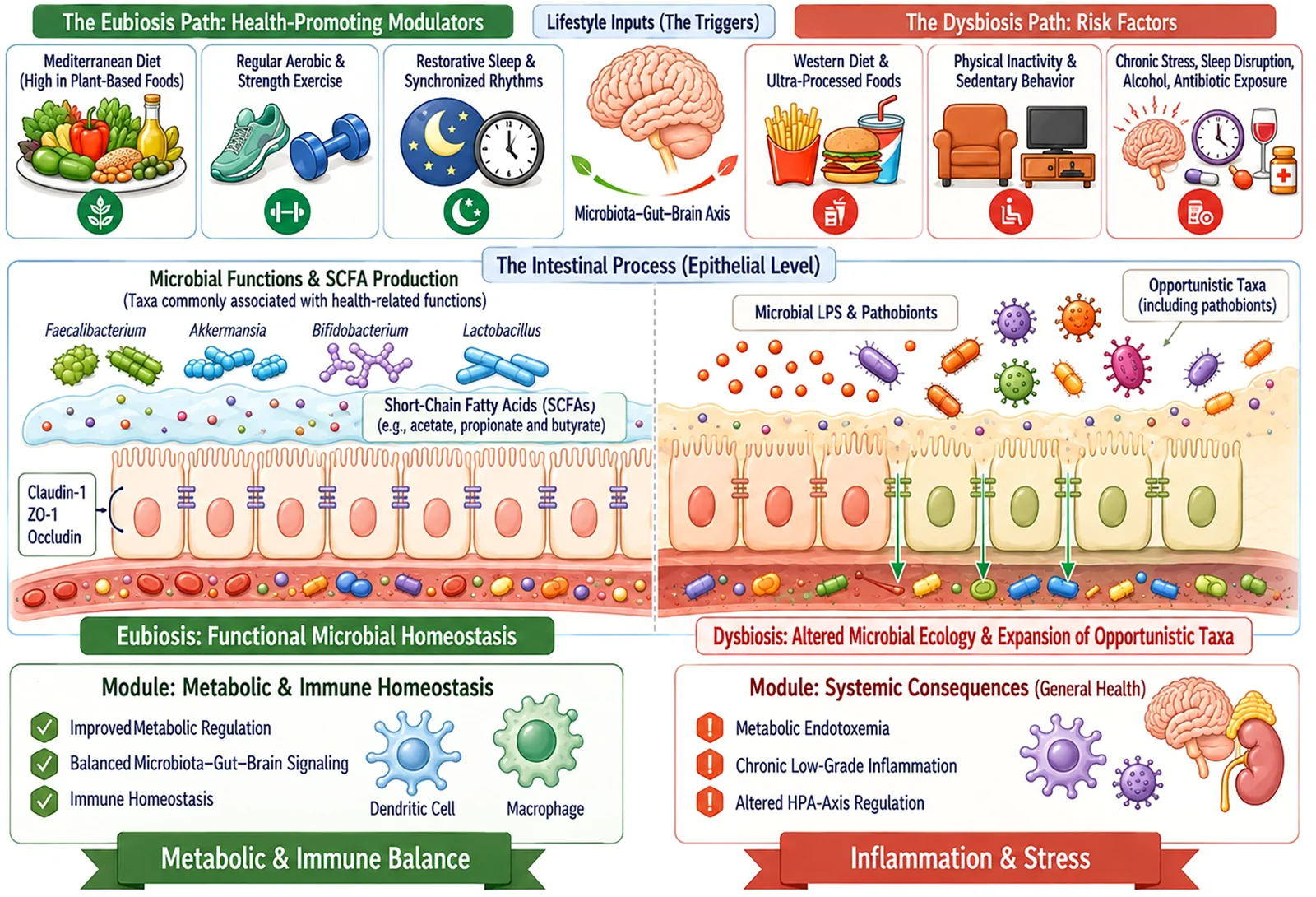
Gut health crossroads: dietary, lifestyle, and environmental modulators of microbial homeostasis. Conceptual framework contrasting health-promoting factors associated with functional microbial homeostasis and risk factors associated with dysbiosis and systemic consequences. HPA, hypothalamic–pituitary–adrenal; LPS, lipopolysaccharide; SCFA, short-chain fatty acid; ZO-1, zonula occludens-1.

Dietary fat strongly influences gut microbial composition and function. However, its effects depend on the amount, type, and duration of fat intake, as well as the overall dietary context. High-fat dietary patterns have been associated with altered microbial diversity and shifts in the relative abundance of specific bacterial taxa, along with changes in microbial metabolites and host inflammatory responses. Although some high-fat diet studies report increases in the Firmicutes-to-Bacteroidetes (F/B) ratio, this ratio should not be considered a universal marker of dysbiosis because findings vary substantially across experimental models, populations, and dietary interventions (Barber et al., 2023; Beam et al., 2021).

Experimental and clinical evidence suggests that high-fat dietary exposure may reduce microbial diversity and alter the abundance of taxa involved in carbohydrate fermentation and short-chain fatty acid production. In a randomized controlled feeding study in healthy adults, higher-fat diets were associated with lower microbial α-diversity, reductions in taxa such as *Faecalibacterium*, and changes in microbial metabolic activity. In contrast, lower-fat diets were associated with greater microbial diversity (Wan et al., 2019). These findings suggest that dietary fat may influence gut microbial ecology not simply through changes in a single bacterial phylum, but through broader effects on community structure and microbial metabolic capacity.

The excessive consumption of refined sugars and sodium that commonly characterizes Western dietary patterns may further influence gut microbial ecology and host metabolic homeostasis. When fructose intake exceeds intestinal absorptive capacity, some fructose may reach the colon, where resident microorganisms can metabolize it and potentially alter microbial composition and metabolite production. However, these effects depend on dose, food matrix, absorptive capacity, and the overall dietary context. Excessive intake of added sugars has also been linked to altered microbial diversity and reduced abundance of some short-chain fatty acid-producing taxa (Jensen et al., 2018).

High dietary sodium represents another potential modulator of the gut microbiota. Experimental studies have reported changes in microbial composition and microbial metabolites in response to high-salt diets, particularly in association with hypertension and immune activation. Nevertheless, these findings largely come from experimental models, and their relevance to human populations requires further investigation (Nova et al., 2022; Kundra et al., 2021).

Dietary changes can rapidly and measurably alter gut microbial composition and function. However, the magnitude and persistence of these changes vary among individuals and depend on the dietary intervention and baseline microbial ecosystem (Sonnenburg et al., 2010). Short-term dietary interventions may alter microbial metabolic pathways before major changes in overall community composition become evident, highlighting the importance of considering both taxonomic and functional responses.

Experimental evidence supports the gut microbiota’s functional contribution to weight regulation. For example, transplanting microbiota from the obese twin of a discordant pair into germ-free mice increased adiposity under specific experimental conditions, showing that microbial communities can influence host metabolic phenotypes (Ridaura et al., 2013). Furthermore, fecal microbiota transplantation from healthy donors into individuals with metabolic syndrome has been reported to improve selected metabolic parameters, although the magnitude and persistence of these effects remain variable (Vrieze et al., 2012).

### 3.3 Key food components

#### 3.3.1 Fiber

Dietary fiber is a major substrate for gut microbial fermentation. It can modulate gut microbiota composition, microbial metabolic activity, and host metabolic regulation, in part through the production of short-chain fatty acids (SCFAs), including acetate, propionate, and butyrate. Although fiber intake is often associated with changes in microbial composition and enrichment of taxa that can utilize complex carbohydrates, the magnitude and direction of these effects vary by fiber type, chemical structure, dose, intervention duration, and baseline microbiota composition (Myhrstad et al., 2020).

Fiber effects are highly substrate-specific, as different chemical structures selectively enrich microorganisms with distinct carbohydrate-degrading capacities and generate divergent metabolic responses. For example, inulin and pectin promote different microbial taxa, illustrating that fiber structure is an important determinant of microbiota response (Chung et al., 2016; Senghor et al., 2018).

Beyond broad taxonomic patterns, dietary fiber exerts substrate- and species-specific effects on the gut microbiota. *In vitro* colonic models have shown that increased fiber intake can stimulate *Bifidobacterium*, *Ruminococcus*, and *Lactobacillus-Enterococcus* groups, as well as *Faecalibacterium prausnitzii* and members of the *Eubacterium rectale–Clostridium coccoides* group (Shen et al., 2012).

Recent 16S rRNA gene sequencing has further resolved this prebiotic specificity at the operational taxonomic unit (OTUs) level, illustrating how distinct non-digestible carbohydrates selectively restructure the microbiome. For example, inulin fermentation strongly enriches two *Bacteroides* OTUs closely related to *Bacteroides uniformis* and *Bacteroides caccae*. In contrast, apple pectin fermentation selectively enriches six distinct OTUs, including Bacteroides *cellulosilyticus*/*intestinalis*, *B. eggerthii*, *B. ovatus*, *B. stercoris*, *B. thetaiotaomicron*, and *B. vulgatus/dorei* (Chung et al., 2016; Senghor et al., 2018).

#### 3.3.2 Polyphenols

Dietary polyphenols are abundant in fruits, vegetables, whole grains, nuts, legumes, tea, coffee, cocoa, and other plant-derived foods and beverages. Their biological effects are closely linked to their interaction with the gut microbiota, which can modify both microbial ecology and polyphenol bioavailability (**Figure 2**) (Du et al., 2021). Researchers have extensively studied both polyphenols and fiber for their ability to modulate the gut microbiota. Specifically, certain polyphenols have been reported to enrich selected intestinal taxa, including *Bifidobacterium*, *Lactobacillus*, *Akkermansia*, and members of the Christensenellaceae family, although these associations are context-dependent (Wu et al., 2021). Although studies often examine individual polyphenols, microbiota-mediated responses vary with compound, dose, bioavailability, food matrix, and microbial metabolic capacity, while real-world intake involves diverse food combinations. A key feature of polyphenol–microbiota interactions is metabolic convergence: structurally distinct dietary polyphenols may be transformed by microbial communities into overlapping or structurally related metabolites, contributing to common biological effects despite differences in the parent compounds (Zamora-Ros et al., 2016).

**Figure 2:**
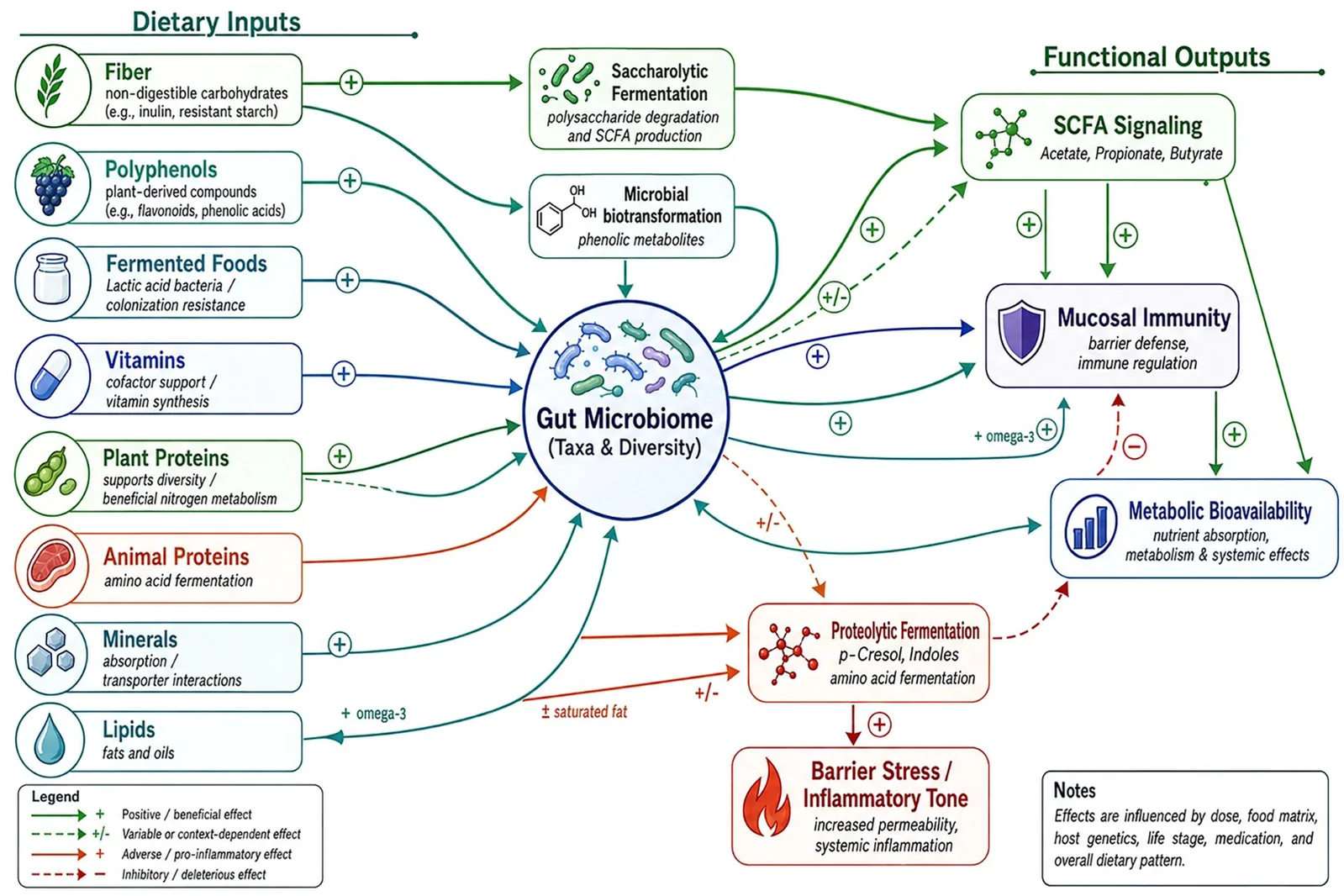
Mechanistic interaction of key food components on microbiota. Eight distinct classes of dietary input–namely fiber, polyphenols, fermented foods, vitamins, plant proteins, animal proteins, minerals, and lipids–converge upon a central microbiome hub, collectively dictating microbial community structure, ecological diversity, functional genomics, and downstream metabolite production.

Poorly absorbed in the upper gut, dietary polyphenols undergo extensive colonic biotransformation by the microbiota (Astorga-Gaxiola et al., 2023). Resveratrol, a plant defense polyphenol, interacts bidirectionally with gut microbes: it modulates microbial composition, while the microbiota activates resveratrol precursors to enhance its bioavailability. In high-fat diet-fed animal models, resveratrol supplementation has been associated with changes in microbial community structure, intestinal barrier function, and metabolic phenotypes. Some studies have reported reductions in the F/B ratio alongside enrichment of Lactobacillus and Bifidobacterium; however, these taxonomic changes should be interpreted within the specific experimental context rather than as universal markers of microbiota improvement (Du et al., 2021).

Flavan-3-ols are highly abundant in tea, nuts, and berries, occurring as both monomers (known as catechins) and polymers (Proanthocyanidins) (Marquez Campos et al., 2020; Pei et al., 2020). In the colon, the gut microbiota biotransform these compounds into derivatives of phenyl-γ-valerolactone and phenylvaleric acid. Similarly, dietary anthocyanin consumption has been shown to shift the microbial profile by enriching the Bacteroidetes phylum and increasing short-chain fatty acid (SCFA) production while reducing Firmicutes abundance (Verediano et al., 2021).

Polyphenol-rich foods and beverages, including red wine and tea, have been associated with shifts in gut microbial composition and increases in taxa linked to short-chain fatty acid production, although the magnitude and direction of these effects depend on the specific polyphenol profile and dietary context (Moreno-Indias et al., 2016; Henning et al., 2018). While research often focuses on individual polyphenols, dietary exposure typically involves complex food matrices containing multiple compounds. These compounds can undergo overlapping microbial biotransformation pathways, generating structurally related metabolites and contributing to shared biological effects (Eseberri et al., 2022).

Dietary polyphenols, ingested as glycosides or aglycones, follow highly characterized metabolic pathways. Lactase-phlorizin hydrolase (LPH) or cytosolic beta-glucosidases hydrolyze glycosides into absorbable aglycones, while unabsorbed compounds undergo colonic microbial fermentation (Eseberri et al., 2022). Once absorbed, they undergo rapid intestinal and hepatic Phase I (Cytochrome P450) and Phase II (UGTs, COMT, sulfotransferases) metabolism. This enzymatic activity varies widely and is influenced by age, genetics, and health status (Eker et al., 2020).

#### 3.3.3 Fermented foods and probiotics

Fermented foods are increasingly recognized as dietary matrices that can influence the gut microbiota through live microorganisms, fermentation-derived metabolites, and changes in food composition and bioavailability. Fermentation can modify carbohydrates, proteins, lipids, and phytochemicals, generating organic acids, bioactive peptides, vitamins, and other microbial metabolites that may interact with the intestinal ecosystem. However, the microbial composition and biological effects of fermented foods depend heavily on the food matrix, fermentation substrate, microorganisms involved, processing conditions, and storage practices. Consequently, fermented food consumption should not be equated with probiotic administration, because only specific microbial strains that meet established criteria and have demonstrated health benefits can be classified as probiotics (Hill et al., 2014; Chandrasekaran et al., 2024).

Probiotics are defined as “live microorganisms that, when administered in adequate amounts, confer a health benefit on the host” (Hill et al., 2014). This definition emphasizes that probiotic effects are microorganism- and strain-specific and require evidence of a beneficial effect under a defined condition of use. Commercially relevant probiotic microorganisms include members of *Lactobacillaceae* and *Bifidobacteriaceae*, as well as selected strains from other genera, depending on the intended application (Chandrasekaran et al., 2024). Importantly, the taxonomy of several traditional probiotic organisms has been revised in recent years; therefore, probiotic identification should preferably be reported at the strain level rather than relying exclusively on genus-level classification.

Probiotic microorganisms may influence host physiology through complementary mechanisms, including modulating intestinal barrier integrity, competing with potentially pathogenic microorganisms, producing antimicrobial compounds, modifying the intestinal luminal environment, and regulating innate and adaptive immune responses (Maldonado Galdeano et al., 2019; Thoda and Touraki, 2023). Certain probiotic strains also produce antimicrobial metabolites, including bacteriocins, organic acids, hydrogen peroxide, and other compounds that can modify microbial interactions and inhibit the growth of competing microorganisms (Bermudez-Brito et al., 2012; Sakandar and Zhang, 2021).

Beyond local intestinal effects, researchers have investigated probiotic interventions for their potential to influence metabolic and inflammatory outcomes. Meta- analysis and clinical studies have reported modest improvements in selected metabolic markers, including fasting glucose and glycated hemoglobin, in some populations with metabolic disorders; however, the magnitude and consistency of these effects vary substantially according to the probiotic strain, dose, duration of intervention, and characteristics of the host (Hsieh et al., 2018; Hasain et al., 2022; Chandrasekaran et al., 2024).

Preclinical studies further suggest that probiotic supplementation may influence glucose homeostasis through interactions with the gut microbiota and enteroendocrine signaling. For example, administration of composite probiotics to diabetic *db/db* mice was associated with alterations in gut microbial composition and increased GLP-1-related signaling (Wang et al., 2020). These findings provide mechanistic support for a microbiota–gut hormone connection, although translating these effects to humans requires further clinical validation.

Strain-specific effects have also been reported for individual probiotic preparations, highlighting the importance of strain-level characterization when evaluating probiotic efficacy and cautioning against extrapolating findings from specific strains to entire genera or species (McFarland, 2021).

Probiotic effects are context-dependent and may vary according to strain, dose, formulation, treatment duration, baseline microbiota, diet, medication exposure, and host health status. Consequently, probiotic supplementation should not be considered a universal strategy for correcting dysbiosis, and safety and efficacy should be evaluated in the specific context of use (Rocha-Ramírez et al., 2017; Chandrasekaran et al., 2024).

#### 3.3.4 Vitamins

Vitamins participate in a bidirectional relationship with the gut microbiota, influencing microbial growth, metabolic activity, and host immune responses. At the same time, intestinal microorganisms contribute to the synthesis and transformation of several vitamins. The magnitude and direction of these interactions depend on the specific vitamin, its chemical form and dietary availability, host nutritional status, and the metabolic capacity of the resident microbiota (Pham et al., 2021; Zhai et al., 2022).

The gut microbiota contributes to the biosynthesis of vitamin K and several B vitamins, including biotin, folate, niacin, riboflavin, thiamine, and pyridoxine. However, the extent to which microbially produced vitamins contribute directly to host nutritional requirements varies substantially by vitamin and physiological condition. Microbial biosynthetic capacity does not necessarily translate into equivalent systemic availability for the host, as vitamin production, microbial uptake, intestinal location, and host absorption can differ considerably among nutrients (Steinert et al., 2020; Zhai et al., 2022).

Vitamin availability may also shape microbial ecology because vitamins function as essential cofactors for numerous microbial metabolic pathways. Consequently, vitamin availability within the intestinal environment can influence microbial growth and metabolic interactions, while microbial metabolism can modify the availability and chemical forms of dietary or host-derived micronutrients. This reciprocal relationship illustrates that vitamins should not be considered exclusively as nutrients for the host but also as components of the metabolic environment in which microbial communities develop (Steinert et al., 2020; Pham et al., 2021).

Among the vitamins investigated in relation to the gut microbiota, vitamin D has received particular attention because of its effects on intestinal barrier integrity, antimicrobial defense, and immune regulation. Vitamin D signaling through the vitamin D receptor (VDR) helps maintain epithelial integrity and regulates antimicrobial peptides and immune responses, thereby influencing interactions between the intestinal mucosa and resident microorganisms (Fakhoury et al., 2020; Tangestani et al., 2021).

Experimental and observational studies have also linked vitamin D status to differences in gut microbial composition and markers of intestinal inflammation. However, these associations should be interpreted cautiously because vitamin D status is closely related to other factors, including diet, adiposity, physical activity, season, and overall health status. Furthermore, evidence on the effects of vitamin D supplementation on gut microbiota composition remains heterogeneous, and the mechanisms linking vitamin D status to specific microbial taxa are not fully established (Fakhoury et al., 2020; Tangestani et al., 2021).

Other vitamins may influence host–microbiota interactions by affecting microbial metabolism and mucosal immunity. Vitamins of the B group participate in microbial biosynthetic and metabolic networks, whereas vitamins A, C, and E have been investigated for potential antimicrobial, antioxidant, or immunomodulatory effects in experimental systems (Kallio et al., 2012; Castillo et al., 2016; Pham et al., 2021).

Nevertheless, evidence for direct antimicrobial effects should be distinguished from physiological effects resulting from habitual dietary intake, because concentrations used in experimental systems may not reflect those achieved in the intestinal lumen following normal food consumption. Therefore, interpret microbiota-modulating effects attributed to individual vitamins according to the experimental model, dose, chemical form, and physiological context in which they were evaluated (Pham et al., 2021).

Vitamin deficiency may further alter the intestinal environment and modify host–microbiota interactions. Conversely, changes in microbial composition and metabolic activity may influence vitamin synthesis, transformation, and availability, making causality difficult to establish in many observational studies (Steinert et al., 2020; Zhai et al., 2022).

Importantly, responses to vitamin intake or supplementation may vary by baseline nutritional status, habitual dietary pattern, host physiology, medication exposure, and the vitamin biosynthetic and metabolic potential of the resident microbiota. This variability highlights the importance of considering both host and microbial characteristics when evaluating micronutrient interventions and may link vitamin–microbiota interactions to personalized nutrition (Pham et al., 2021; Zhai et al., 2022).

#### 3.3.5 Proteins

Dietary proteins can influence the gut microbiota according to their source, quantity, amino acid composition, digestibility, and the proportion reaching the colon (Neis et al., 2015; Yang et al., 2020). Colonic protein provides substrates for microbial proteolytic fermentation, generating metabolites such as ammonia, branched-chain fatty acids, phenols, indoles, p-cresol, and hydrogen sulfide. Their effects are context-dependent: some participate in host–microbiota signaling, whereas others may adversely affect intestinal epithelial physiology at elevated concentrations (Neis et al., 2015; Yang et al., 2020).

Protein source is an important determinant of microbial responses, although its effects are difficult to separate from other dietary components. Animal-based diets have been associated with enrichment of bile-tolerant taxa such as Alistipes, Bilophila, and Bacteroides and reductions in bacteria involved in plant-polysaccharide utilization, including Roseburia, Eubacterium rectale, and Ruminococcus bromii. However, differences in fat and fiber intake limit attribution of these effects to protein alone. Therefore, associations between protein source and gut microbiota should be interpreted within the context of the overall dietary pattern, including fiber availability, fat composition, food processing, and other co-consumed nutrients (David et al., 2014).

Overall, dietary context strongly influences protein–microbiota associations. Bacteroides- and Prevotella-enriched communities have been associated with dietary patterns differing in animal-derived foods and complex carbohydrates, respectively; these findings should therefore not be interpreted as evidence that protein intake alone determines a specific enterotype (Wu et al., 2011; Costea et al., 2018).

Preclinical studies indicate that protein source and composition can influence gut microbial communities. Whey protein and casein supplementation have been associated with changes in Lactobacillus, Bifidobacterium, and other bacterial taxa in experimental models (Sprong et al., 2010; McAllan et al., 2014). However, the translational relevance of these findings should be interpreted cautiously because animal models differ from humans in dietary composition, intestinal physiology, microbial ecology, and protein metabolism.

Plant- and animal-derived proteins may also differ in their effects on microbial metabolism because of differences in amino acid composition, digestibility, food structure, and accompanying dietary components. Experimental studies indicate that cereal and meat proteins can produce distinct fermentation profiles, including differences in ammonia, pH, SCFA production, and specific bacterial groups (Wang et al., 2020). Thus, protein effects should be considered in relation to digestibility, processing, amino acid composition, and other fermentable substrates rather than classified simply as “beneficial” or “harmful” (Yang et al., 2020).

Fermentable carbohydrate availability is another key determinant of protein–microbiota interactions. Greater carbohydrate availability may favor saccharolytic fermentation, whereas increased protein substrates reaching the distal colon may promote proteolytic fermentation and amino acid-derived metabolites. This interaction highlights the importance of considering dietary protein together with fiber and other carbohydrate sources (Diether and Willing, 2019; Yang et al., 2020).

Protein-induced microbial responses also vary among individuals according to baseline microbiota, habitual diet, host metabolic status, and microbial metabolic capacity. Dietary sulfur-containing amino acids, for example, can produce distinct microbial and metabolic responses despite limited population-level changes in selected sulfate-reducing bacteria (Dostal Webster et al., 2019). Overall, protein–microbiota interactions depend on protein source, digestibility, dose, dietary context, and host–microbiota characteristics rather than producing a uniform microbial response (Yang et al., 2020).

#### 3.3.6 Minerals

Minerals and the gut microbiota interact bidirectionally: minerals can influence microbial growth and metabolic activity, while gut microorganisms may affect mineral bioaccessibility and absorption. Calcium, iron, zinc, magnesium, and phosphorus have been associated with changes in microbial composition or function, although responses depend on mineral dose, chemical form, food matrix, host nutritional status, and baseline microbiota (Bielik and Kolisek, 2021; Barone et al., 2022). Microbial metabolism may also influence mineral availability through processes such as phytate degradation and changes in the intestinal physicochemical environment, although its contribution to human mineral status remains incompletely characterized (Bielik and Kolisek, 2021; Barone et al., 2022).

Calcium, phosphorus, and magnesium may influence the intestinal environment through distinct mechanisms. Calcium has been associated with changes in microbial metabolites and specific bacterial groups, whereas the independent effects of phosphorus remain unclear. Magnesium deficiency may alter intestinal permeability and microbial diversity, while supplementation may partially restore microbial richness; however, human evidence remains limited (Gommers et al., 2019; Yang et al., 2020; Kundra et al., 2021; Yoon and Michels, 2021).

Iron represents an important example of host–microbiota competition for a micronutrient. Iron supplementation has been associated with changes in microbial communities, including reductions in Bifidobacterium. It increases Lactobacillus in some pediatric studies, whereas greater luminal iron availability may favor facultative anaerobes such as Enterobacteriaceae under specific conditions. These effects depend on age, iron status, supplementation regimen, and baseline microbiota and should not be directly extrapolated across populations (Zimmermann et al., 2010; Kundra et al., 2021; Mayneris-Perxachs et al., 2022).

Other minerals, including zinc, selenium, manganese, and copper, may influence microbial ecology, redox processes, and intestinal barrier function, although evidence remains heterogeneous (Zackular et al., 2016; Barra et al., 2021; Kundra et al., 2021; Wu et al., 2024). Dietary sodium provides another example: high-salt diets have been associated with changes in selected *Lactobacillus* populations, microbial metabolites, and Th17-related immune signaling, although these effects should be interpreted as consequences of high-salt dietary exposure rather than sodium acting independently (Kundra et al., 2021; Nova et al., 2022).

Overall, mineral–microbiota interactions vary according to mineral status, dietary matrix, supplementation dose, medication use, and baseline microbiota. Further human studies integrating mineral biomarkers, microbiota profiles, and functional outcomes are needed to establish microbiome-targeted mineral interventions (Bielik and Kolisek, 2021; Barone et al., 2022).

#### 3.3.7 Lipids

Dietary lipids influence the gut microbiota according to fatty acid composition, chain length, degree of unsaturation, dose, and dietary context. Conversely, gut microorganisms can modify dietary and host-derived lipids and transform bioactive lipid metabolites, potentially affecting intestinal barrier function, immune signaling, and metabolic homeostasis (Brown et al., 2023; Mohammadi and Rudkowska, 2025).

Dietary fat should be distinguished from fermentable carbohydrates, as microbes primarily generate SCFAs through carbohydrate fermentation rather than lipid metabolism. Nevertheless, dietary fat can indirectly influence SCFA production by altering microbial community structure, intestinal transit, bile acid metabolism, and the availability of fermentable substrates. Microbial lipid metabolism may also generate or modify bioactive compounds, including secondary bile acids and fatty acid-derived metabolites involved in host–microbiota signaling (Agus et al., 2021; Brown et al., 2023).

N-3 polyunsaturated fatty acids (PUFAs), including alpha-linolenic acid (ALA), eicosapentaenoic acid (EPA), and docosahexaenoic acid (DHA), have been investigated for their effects on gut microbial communities and inflammatory pathways. Human and animal studies have investigated the effects of n-3 PUFA supplementation on gut microbial communities, with some studies reporting changes in selected Bifidobacterium and Lactobacillus populations. However, findings are heterogeneous across study populations and experimental models. Animal studies have also reported enrichment of taxa such as Proteobacteria and Desulfovibrio, although the translational relevance of these findings to humans remains uncertain (Bassaganya-Riera and Hontecillas, 2010; Mujico et al., 2013).

Microbial metabolites may mediate some metabolic effects associated with dietary fat. In C57BL/6J mice, sodium butyrate supplementation during a high-fat diet improved glucose intolerance and insulin resistance and altered gut microbial composition, including increased Verrucomicrobia (Gao et al., 2019). However, because this study evaluated direct butyrate supplementation, it supports a role for microbial metabolites in modulating metabolic responses to a high-fat environment rather than a direct lipid-to-butyrate pathway (Gao et al., 2019).

Dietary lipids may also influence host physiology through microbial metabolite and bile acid pathways. Microbial conversion of primary to secondary bile acids can modulate FXR and TGR5 signaling, linking microbial activity with lipid metabolism, glucose homeostasis, and immune regulation (Agus et al., 2021; Brown et al., 2023).

Overall, lipid effects on the gut microbiota depend on fatty acid structure, dietary source, dose, food matrix, accompanying nutrients, host metabolic status, and baseline microbiota. Because much mechanistic evidence derives from experimental models and human findings remain heterogeneous, controlled interventions are needed to distinguish effects of specific lipid classes from those of overall dietary patterns (Brown et al., 2023; Mohammadi and Rudkowska, 2025).

Taken together, evidence across the dietary components discussed above indicates that microbiota responses are highly context-dependent. While changes in microbial metabolites and functional pathways, including SCFAs and other bioactive metabolites, are frequently reported, taxonomic responses are more heterogeneous and vary by dietary component, dose, food matrix, baseline microbiota, and study design. Differences in intervention duration, dietary background, analytical methods, and the relative contribution of observational, experimental, and human intervention evidence remain important limitations when comparing findings across dietary components.

## 4 Lifestyle and behavioral determinants

### 4.1 The importance of the microbiota-gut-brain axis in mental health

The gut microbiota is a metabolically active ecosystem that communicates bidirectionally between the gastrointestinal tract and the central nervous system (CNS) through the microbiota–gut–brain axis (MGBA). This communication involves interconnected neural, endocrine, immune, and metabolic pathways that regulate gastrointestinal physiology, stress responses, cognition, and emotional processes (Skonieczna-Zydecka et al., 2020; Cooke et al., 2022). Rather than functioning as an isolated pathway, the MGBA integrates signals originating from the intestinal microbiota, intestinal epithelium, immune system, enteric nervous system, and CNS, providing a biological framework through which gastrointestinal and psychological states can influence one another.

Several mechanisms may mediate communication between gut microorganisms and the brain. Microorganisms can produce or transform neuroactive compounds and microbial metabolites, including short-chain fatty acids (SCFAs), tryptophan-derived metabolites, and other signaling molecules that can influence immune, endocrine, and neuronal pathways. The vagus nerve provides an important neural communication route, while microbial metabolites and immune mediators can act through peripheral circulation and influence neuroimmune and neuroendocrine processes (**Table 2**) (Skonieczna-Zydecka et al., 2020; Cooke et al., 2022). The intestinal barrier and the blood–brain barrier further shape this communication by regulating host tissue exposure to microbial products and circulating metabolites.

**Table 2:** Gut microbiota alterations reported in mental disorders and potential mechanisms.

Microbial taxon	Reported mental health association	Evidence level	Potential mechanisms/microbial functions	Potential host signaling pathways	References
*Faecalibacterium* (*F. prausnitzii*)	Reduced abundance reported in MDD, bipolar disorder, and schizophrenia	Human observational; mechanistic evidence	Major butyrate producer; may contribute to intestinal barrier integrity and immune regulation.	SCFA-mediated modulation of immune and neuroinflammatory pathways.	(Nikolova et al., 2021)
*Coprococcus* (*C. catus*)	Reduced abundance reported in depression and anxiety; associations with mental well-being	Human observational; mechanistic evidence	SCFA production and potential contribution to intestinal epithelial and immune homeostasis.	Indirect effects through SCFA-related signaling	(Skonieczna-Zydecka et al., 2020)
*Eggerthella*	Increased abundance reported in some psychiatric disorder cohorts	Human observational	Associated with altered microbial metabolic activity and inflammatory profiles	Potential contribution to systemic inflammatory signaling	(Nikolova et al., 2021)
*Lactobacillus*	Strain- and population-specific associations with mood and anxiety	Human observational; mechanistic evidence	Some strains can produce or modulate neuroactive compounds and interact with immune and vagal pathways.	GABA-related and vagal signaling; effects are strain-specific	(Skonieczna-Zydecka et al., 2020)
*Bifidobacterium*	Associations with lower anxiety and depressive symptoms reported in some studies.	Human observational; mechanistic evidence	Production of microbial metabolites; contribution to intestinal barrier and immune regulation	Potential modulation of stress responsiveness and gut–brain signaling	(Skonieczna-Zydecka et al., 2020)
*Bacteroides*	Altered abundance reported in depressive phenotypes; direction varies among studies	Human observational	Species-dependent effects on amino acid and carbohydrate metabolism	Potential modulation of GABA- and tryptophan-related pathways	(Skonieczna-Zydecka et al., 2020)
*Lachnospiraceae/Ruminococcaceae*	Reduced abundance reported in some MDD cohorts	Human observational; animal experimental; mechanistic evidence	Include several SCFA-producing taxa and contribute to carbohydrate fermentation	Potential effects on intestinal barrier, immune regulation, and neuroimmune signaling	(Nikolova et al., 2021)
Enterobacteriaceae/ *Escherichia-Shigella*	Increased abundance reported in some psychosis, bipolar disorder, and anxiety cohorts	Human observational; mechanistic evidence	Includes taxa capable of producing LPS and other microbial metabolites	Potential activation of inflammatory and TLR4-associated signaling	(Nikolova et al., 2021)
*Prevotella*	Associations reported in some schizophrenia cohorts; population-dependent	Human observational	Involved in carbohydrate metabolism and production of microbial metabolites	Potential effects on SCFA- and tryptophan-related pathways	(Skonieczna-Zydecka et al., 2020)
*Klebsiella, Proteus, Morganella*	Increased abundance reported in some dysbiotic states associated with psychiatric disorders	Human observational	Production of biogenic amines and other microbial metabolites	Potential effects through inflammatory and metabolic signaling	(Skonieczna-Zydecka et al., 2020)

MDD, major depressive disorder; SCFA, short-chain fatty acid; GABA, γ-aminobutyric acid; LPS, lipopolysaccharide; TLR4, Toll-like receptor 4. Taxonomic associations represent findings reported across studies and should not be interpreted as disease-specific microbial signatures or evidence of causality. Evidence levels indicate the predominant type of evidence supporting the reported association and do not represent a formal assessment of evidence quality. Proposed mechanisms are based on experimental and observational evidence and may vary according to microbial strain, host characteristics, disease status, medication use, diet, and study design.

The microbiota may also interact with the hypothalamic–pituitary–adrenal (HPA) axis, a key component of the physiological stress response. Experimental and clinical evidence suggests that stress can alter intestinal microbial communities, while microbial perturbations may influence stress-related neuroendocrine responses. This bidirectional relationship may explain how chronic psychological stress and changes in gut microbial ecology reinforce one another (Cooke et al., 2022; Marques et al., 2023). However, the extent to which specific microbial changes directly contribute to human psychological disorders remains incompletely established.

#### 4.1.1 Gut microbiota alterations in mental disorders

Alterations in gut microbial composition have been reported in individuals with several psychiatric disorders, including major depressive disorder (MDD), anxiety disorders, bipolar disorder, and schizophrenia. However, microbial signatures vary across studies, and differences in diet, medication use, age, sex, disease severity, lifestyle, geographical location, and methodological approaches can contribute to heterogeneous findings (Skonieczna-Zydecka et al., 2020; Nikolova et al., 2021). Consequently, currently reported microbial alterations should be interpreted as disease-associated patterns rather than definitive microbial signatures of specific psychiatric disorders.

Some cross-diagnostic patterns have nevertheless been reported. For example, reductions in taxa involved in SCFA production, including members of *Faecalibacterium* and *Coprococcus*, and alterations in potentially pro-inflammatory taxa have been described in individuals with psychiatric conditions (Skonieczna-Zydecka et al., 2020; Nikolova et al., 2021). These observations are biologically relevant because changes in microbial metabolic capacity may influence intestinal barrier function, immune signaling, and the availability of metabolites involved in host–microbiota communication; however, taxonomic changes alone do not establish whether microbial alterations cause, result from, or modify psychiatric disease.

Altered microbial metabolism may provide a mechanistic link between intestinal microbial communities and brain function. Changes in SCFA production may affect epithelial integrity and immune regulation, whereas microbial metabolism of tryptophan and other amino acids can generate compounds that interact with host signaling pathways. In addition, microbial and host metabolism of neurotransmitter precursors, including pathways related to γ-aminobutyric acid (GABA), glutamate, and tryptophan, may contribute to neurochemical signaling (Cooke et al., 2022; Skonieczna-Zydecka et al., 2020). Nevertheless, the contribution of these pathways to human psychiatric disease remains an active area of investigation, and mechanistic evidence is stronger in experimental models than in clinical populations.

An additional mechanism involves intestinal permeability and immune activation. Alterations in microbial communities may influence epithelial barrier function and exposure to microbial-associated molecular patterns such as lipopolysaccharide (LPS), potentially promoting systemic inflammatory signaling. Cytokines and other immune mediators may subsequently affect neuroimmune communication and brain function, providing a plausible link between intestinal microbial disturbances and neuroinflammatory processes (Skonieczna-Zydecka et al., 2020; Cooke et al., 2022). However, the relationship between dysbiosis, intestinal permeability, inflammation, and psychiatric symptoms is complex and should not be interpreted as a single linear causal pathway.

Overall, current evidence supports the microbiota–gut–brain axis as a biologically plausible framework for understanding interactions between intestinal microbial ecology, stress physiology, and mental health. Still, important uncertainties remain regarding causality and clinical translation. Future studies should prioritize longitudinal human cohorts, standardized microbiome methodologies, integration of dietary and medication data, and multi-omics approaches capable of linking microbial functions with host metabolic, immune, and neurobiological outcomes. Such approaches may help distinguish disease-associated microbial changes from causal mechanisms and identify individuals who are more likely to respond to microbiota-targeted interventions.

### 4.2 Exercise and physical activity

Physical activity is an important lifestyle factor influencing gut microbial ecology and function. Beyond its established benefits for cardiovascular, metabolic, musculoskeletal, and mental health, regular exercise may modify intestinal microbial communities through changes in gastrointestinal motility, epithelial barrier function, immune regulation, bile acid metabolism, substrate availability, and microbial metabolite production (Aragón-Vela et al., 2021; Campaniello et al., 2022).

Although diet remains a major determinant of gut microbiota composition, sustained physical activity may also affect microbial diversity, community structure, and metabolic activity. However, these effects are heterogeneous, and distinguishing the independent contribution of exercise from dietary and other lifestyle factors remains challenging because physically active individuals often differ from sedentary populations in dietary quality, body composition, and metabolic health (Campaniello et al., 2022; Min et al., 2024).

Recent systematic reviews indicate that physical activity is often associated with increased abundance of microorganisms involved in SCFA production and other metabolic functions, including Akkermansia, Faecalibacterium, Roseburia, and Veillonella. Nevertheless, findings regarding α-diversity and specific taxonomic changes remain inconsistent, reflecting differences in exercise modality, intensity, duration, participant characteristics, dietary control, and microbiome profiling methods (Pérez-Prieto et al., 2024). Thus, exercise may influence selected microbial functions and community features without necessarily increasing overall microbial diversity.

Importantly, the relationship between exercise and the gut microbiota is dose- and context-dependent. Regular physical activity may support intestinal barrier homeostasis, whereas acute strenuous exercise can transiently increase intestinal permeability. These responses depend on exercise intensity, duration, training status, and individual physiological characteristics (Keirns et al., 2020; Campaniello et al., 2022) (**Table 3**).

**Table 3:** Effects of physical activity on gut microbiota and associated host outcomes.

Exercise characteristic	Reported microbial response	Potential microbial functions/metabolites	Associated host outcomes	References
Regular moderate aerobic exercise	Changes in selected SCFA-producing and health-associated taxa; responses heterogeneous	SCFA production and microbial metabolic activity	Intestinal and metabolic homeostasis	(Aragón-Vela et al., 2021; Campaniello et al., 2022)
Resistance training	Changes in selected microbial taxa; evidence more limited	Potential effects on microbial metabolism	Metabolic and musculoskeletal health	(Campaniello et al., 2022)
High-intensity/prolonged exercise	Context-dependent microbial alterations; possible transient perturbation of gut function	Changes in microbial metabolites and intestinal permeability	Potential gastrointestinal stress and inflammatory responses	(Campaniello et al., 2022)
Combined/regular training	Changes in community composition and selected functional taxa	SCFA-related and other microbial metabolic pathways	Potential improvements in metabolic regulation	(Aragón-Vela et al., 2021; Cataldi et al., 2022)

#### 4.2.1 Exercise as a modulator of gut microbial ecology

Exercise can modify the gut microbial ecosystem by altering intestinal transit, luminal substrate availability, host metabolism, immune activity, and gastrointestinal physiology. Observational and intervention studies have reported differences in microbial composition between physically active and sedentary individuals, including enrichment of taxa associated with carbohydrate fermentation and SCFA production (Aragón-Vela et al., 2021; Campaniello et al., 2022).

Taxa frequently associated with physical activity include Akkermansia, Faecalibacterium, Roseburia, and Veillonella, although responses vary across studies. A recent systematic review and meta-analysis reported higher relative abundance of several SCFA-producing taxa in physically active populations, while highlighting substantial heterogeneity. Similarly, evidence for exercise-associated changes in Akkermansia muciniphila differs between human and animal studies (Pérez-Prieto et al., 2024).

Effects on microbial diversity are less consistent than changes in specific taxa or microbial functions. In a recent systematic review, more than half of the studies reported no significant effect of exercise on microbial diversity. In contrast, studies that reported changes often noted alterations in the Shannon index or β-diversity. Thus, exercise-induced microbial remodeling may involve changes in community structure or functional potential rather than a generalized increase in diversity (Pérez-Prieto et al., 2024).

Exercise characteristics influence the microbial response. Human intervention studies suggest that moderate- to high-intensity exercise performed for approximately 30–90 min at least three times per week, or 150–270 min per week, for eight weeks or longer can produce measurable changes in gut microbiota composition. However, researchers have not established a universal optimal exercise prescription for microbiota modulation, and responses vary by exercise type, training status, age, sex, diet, and baseline microbiota (Campaniello et al., 2022; Min et al., 2024).

Exercise-induced microbial changes should therefore not be interpreted simply as the selection of “beneficial” or “harmful” microorganisms. Rather, physical activity may modify microbial community structure and metabolic capacity within a broader host–diet–microbiota context, with responses influenced by individual physiological and microbial characteristics (Campaniello et al., 2022; Pérez-Prieto et al., 2024).

#### 4.2.2 Exercise, gastrointestinal physiology, and intestinal barrier function

The gastrointestinal tract is both a target and mediator of exercise-induced physiological adaptations. Physical activity can influence intestinal motility, transit time, splanchnic blood flow, body temperature, oxygen availability, and gastrointestinal mechanical stress, thereby modifying the environment in which gut microorganisms reside. These changes may affect microbial substrate availability and, consequently, microbial metabolism and community structure (Campaniello et al., 2022).

Intestinal barrier integrity represents an important interface between exercise, the microbiota, and host immunity. Mucus, epithelial cells, antimicrobial factors, and tight-junction proteins, including occludin and claudins, maintain epithelial barrier function. Regular physical activity may support barrier homeostasis, whereas acute strenuous exercise can transiently disrupt epithelial integrity (Keirns et al., 2020).

These responses depend on exercise intensity and duration. Acute, prolonged, or high-intensity exercise can increase intestinal permeability, partly through reduced splanchnic blood flow, hyperthermia, oxidative stress, and altered tight-junction regulation. Increased permeability may enhance host exposure to luminal microbial products, including lipopolysaccharide (LPS), potentially contributing to transient inflammatory responses (Chantler et al., 2021).

In contrast, repeated exercise training may promote gastrointestinal adaptation and improve intestinal integrity. Chronic aerobic exercise may attenuate some adverse effects observed after acute exercise, although adaptations vary according to training characteristics and individual physiological responses (Keirns et al., 2020).

The interaction between the intestinal barrier and microbiota is bidirectional: microbial metabolites can influence epithelial integrity and immune signaling. In contrast, changes in the intestinal environment can alter microbial community structure and activity. Thus, exercise-induced changes in barrier function may contribute to broader host–microbiota communication (Campaniello et al., 2022).

#### 4.2.3 Exercise, microbial metabolism, and host health

Changes in microbial metabolism represent an important mechanism through which physical activity may influence host physiology. Exercise-associated remodeling of the gut microbiota can alter the production and transformation of microbial metabolites, particularly short-chain fatty acids (SCFAs), bile acid derivatives, and other compounds involved in host–microbiota communication (Aragón-Vela et al., 2021; Campaniello et al., 2022).

SCFAs, including acetate, propionate, and butyrate, are among the microbial metabolites most frequently investigated in relation to physical activity. Exercise has been associated with increased abundance of microorganisms involved in carbohydrate fermentation and SCFA production, although changes in fecal SCFA concentrations are not consistently observed. SCFAs can influence intestinal epithelial integrity, immune regulation, energy metabolism, and host signaling through G protein-coupled receptors and other molecular pathways (Campaniello et al., 2022; Cataldi et al., 2022).

Exercise may also influence microbial bile acid transformation, providing another link between the gut microbiota and systemic metabolism. Microbial conversion of primary into secondary bile acids can modify signaling through host receptors such as the farnesoid X receptor (FXR) and Takeda G protein-coupled receptor 5 (TGR5), which participate in glucose and lipid metabolism, energy homeostasis, and immune regulation. However, the extent to which exercise directly modifies these microbial pathways in humans remains incompletely characterized (Campaniello et al., 2022).

Exercise–microbiota interactions may be particularly relevant to metabolic health (**Figure 3**). Physically active individuals and intervention studies involving obesity or metabolic dysfunction have reported changes in microbial composition and metabolite production, with some studies associating these changes with improved glucose regulation, reduced inflammatory signaling, and enhanced metabolic homeostasis (Aragón-Vela et al., 2021; Campaniello et al., 2022). Nevertheless, microbiota alterations should not be considered the sole explanation for exercise’s metabolic benefits, which also result from direct effects on skeletal muscle, adipose tissue, insulin sensitivity, energy expenditure, and systemic inflammation.

**Figure 3:**
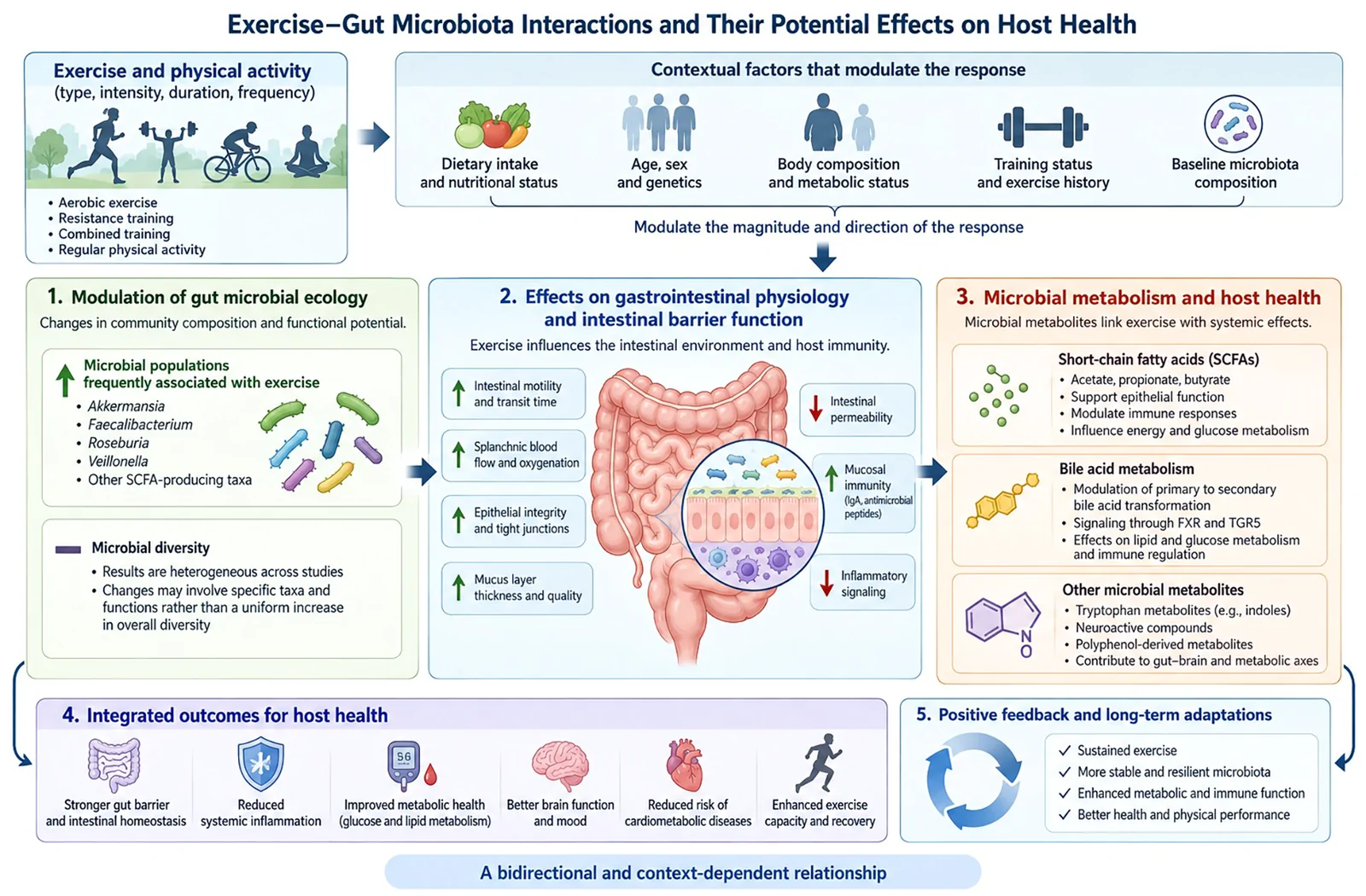
Exercise–gut microbiota interactions and their potential effects on host health. Schematic overview of how physical activity influences gut microbial ecology, gastrointestinal physiology, intestinal barrier function, and microbial metabolism, with responses modulated by host, dietary, and exercise-related factors.

Microbial metabolic responses to exercise are highly context-dependent. Exercise modality, intensity, duration, nutritional status, training status, host metabolic phenotype, and baseline microbiota may influence changes in microbial metabolites. Thus, physical activity may modify specific microbial pathways rather than induce a uniform metabolic profile within an individualized host–diet–microbiota context (Campaniello et al., 2022; Cataldi et al., 2022).

### 4.3 Sleep and circadian rhythms

Sleep and circadian rhythms regulate gastrointestinal physiology and gut microbial activity. The gut microbiota shows temporal fluctuations in microbial abundance, gene expression, and metabolic activity influenced by host circadian rhythms and behavioral cues, particularly the timing of food intake and the sleep–wake cycle (Matenchuk et al., 2020; Lotti et al., 2023). Consequently, alterations in sleep, meal timing, or circadian alignment may modify the temporal organization of host–microbiota interactions and contribute to metabolic and immune dysregulation.

#### 4.3.1 Circadian regulation of the gut microbiota

The gut microbiota exhibits diurnal fluctuations in composition and metabolic activity that are coordinated with the host circadian system. Environmental and behavioral signals, including the light–dark cycle, feeding–fasting patterns, and sleep–wake cycles, influence these rhythms, positioning the microbiota as both a target and a potential mediator of circadian physiology (Matenchuk et al., 2020; Lotti et al., 2023).

The central clock in the suprachiasmatic nucleus (SCN) coordinates host circadian rhythms, while peripheral clocks distributed throughout metabolic tissues, including the gastrointestinal tract, also contribute. At the molecular level, clock genes such as *CLOCK*, *BMAL1*, *PER1–3*, and *CRY1–2* regulate daily oscillations in metabolic, immune, and gastrointestinal processes. Whereas the central clock is primarily entrained by light, peripheral clocks are strongly influenced by feeding–fasting cycles, making meal timing an important regulator of intestinal circadian physiology (Lotti et al., 2023).

Feeding behavior appears to be an important determinant of microbial rhythmicity. Temporal changes in microbial communities have been reported across the day, together with rhythmic variation in microbial metabolic pathways and metabolites. These fluctuations include changes in pathways related to short-chain fatty acids (SCFAs), secondary bile acids, and tryptophan-derived metabolites, which can participate in epithelial, immune, metabolic, and circadian signaling (Matenchuk et al., 2020; Lotti et al., 2023).

The relationship between the host circadian system and the gut microbiota is bidirectional. Circadian signals influence intestinal physiology, feeding behavior, and nutrient availability, thereby shaping microbial activity, whereas microbial metabolites can influence host metabolic and immune pathways and may interact with peripheral clock mechanisms (Matenchuk et al., 2020; Lotti et al., 2023). Thus, microbial rhythmicity represents an important component of the temporal organization of host–microbiota interactions, although the relative contribution of microbial signals to human circadian physiology remains incompletely characterized.

#### 4.3.2 Circadian disruption and gut microbiota alterations

Modern lifestyles can disrupt circadian organization through insufficient or irregular sleep, shift work, jet lag, irregular meal timing, and nighttime light exposure. These conditions may impair synchronization between central and peripheral clocks and alter the temporal organization of gastrointestinal physiology and microbial activity (Lotti et al., 2023; Bautista et al., 2025) (**Table 4**).

**Table 4:** Circadian disruption, gut microbiota alterations, and reported host consequences.

Circadian factor	Gut microbiota alterations	Proposed mechanisms	Physiological consequences	References
Sleep deprivation	Reduced microbial diversity; decreased *Lactobacillus* and SCFA production	HPA-axis activation, increased intestinal permeability	Low-grade inflammation, metabolic dysfunction	(Matenchuk et al., 2020; Yang et al., 2023)
Shift work	Loss of microbial rhythmicity and altered taxonomic composition	Circadian misalignment and irregular feeding	Obesity, insulin resistance	(Zheng et al., 2020; Lotti et al., 2023)
Jet lag	Disrupted diurnal microbial oscillations	Desynchronization between central and peripheral clocks	Glucose intolerance and inflammation	(Zheng et al., 2020; Lotti et al., 2023)
Night-time eating	Reduced abundance of SCFA-producing bacteria	Altered feeding–fasting cycles	Metabolic dysregulation	(Zheng et al., 2020; Lotti et al., 2023)

Reported associations and proposed mechanisms are summarized from observational and experimental evidence and should not be interpreted as evidence of a uniform causal pathway.

Circadian misalignment may initially affect the rhythmicity of microbial activity rather than producing immediate and uniform changes in microbial composition. Studies have reported alterations in the timing of microbial abundance, gene expression, and metabolite production associated with disrupted feeding–fasting cycles and sleep patterns. These changes may precede or accompany broader alterations in microbial community structure and host metabolic regulation (Lotti et al., 2023; Bautista et al., 2025).

Sleep deprivation provides an important example of the interaction between sleep and gut microbial homeostasis. Experimental and human studies have associated insufficient sleep with changes in microbial diversity, selected microbial taxa, and microbial metabolite production, although findings are heterogeneous. In experimental models, sleep deprivation has been associated with alterations in SCFA production, circadian clock gene expression, inflammatory signaling, and intestinal barrier integrity (Matenchuk et al., 2020; Yang et al., 2023).

Circadian disruption may also affect intestinal barrier function and immune signaling. Changes in autonomic and hypothalamic–pituitary–adrenal (HPA) axis activity, along with changes in intestinal physiology, may increase epithelial permeability and alter host exposure to microbial products such as lipopolysaccharide (LPS). These processes provide a plausible link between circadian misalignment, microbial dysregulation, and systemic inflammatory responses (Matenchuk et al., 2020; Yang et al., 2023).

Disruption of microbial rhythmicity may further influence the temporal production of microbial metabolites, including SCFAs, secondary bile acids, and indole derivatives. Because these metabolites participate in host metabolic and immune signaling, altered production may contribute to impaired glucose and lipid metabolism and other metabolic consequences associated with chronic circadian disruption (Lotti et al., 2023).

Nevertheless, evidence from human intervention studies remains limited and heterogeneous. For example, short-term sleep extension in chronically sleep-deprived adults did not significantly alter gut microbial diversity or composition, suggesting that improving sleep alone may not be sufficient to restore microbial homeostasis rapidly (Reutrakul et al., 2020). These findings emphasize that sleep, feeding behavior, circadian alignment, and the gut microbiota should be considered as interconnected factors rather than independent determinants.

#### 4.3.3 Restoring circadian homeostasis through chrononutrition

Circadian alignment depends not only on sleep duration and regularity but also on the timing of food intake. Chrononutrition refers to the interaction between meal timing, meal frequency, feeding–fasting cycles, and the circadian system, recognizing food intake as an important zeitgeber for peripheral clocks in the gastrointestinal tract and other metabolic tissues (Matenchuk et al., 2020; Bajaj and Sharma, 2025).

The gut microbiota may participate in this relationship because feeding–fasting cycles influence nutrient availability, microbial activity, and the temporal organization of microbial metabolism. Conversely, irregular meal timing, nighttime eating, and prolonged feeding windows may alter microbial rhythmicity and host metabolic responses (Lotti et al., 2023; Bajaj and Sharma, 2025; Bautista et al., 2025) (**Figure 4**).

**Figure 4:**
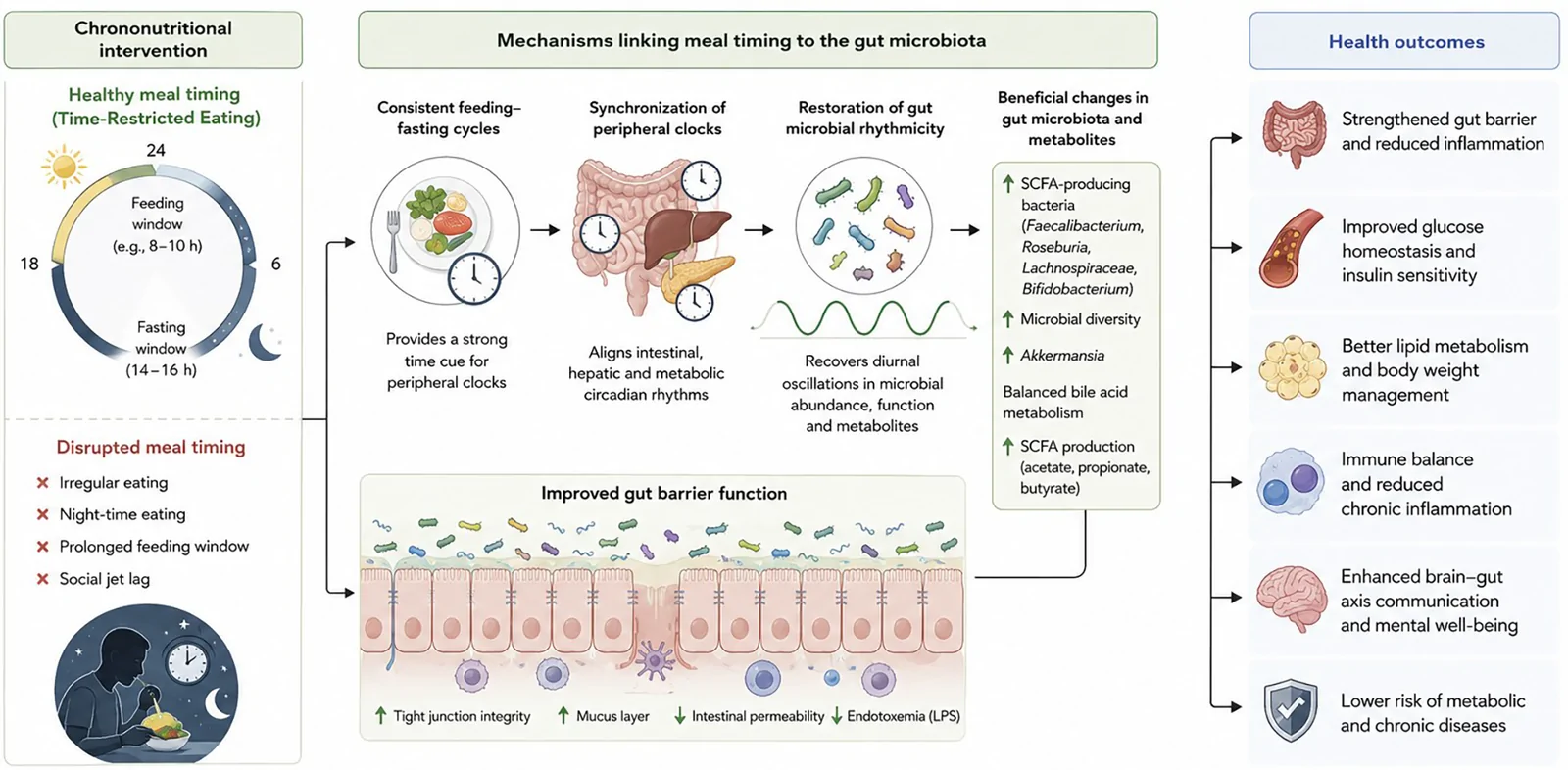
Chrononutrition and gut microbial rhythmicity: potential pathways linking meal timing with host health. Schematic overview of how meal timing and feeding–fasting cycles interact with circadian rhythms and gut microbial activity, with potential effects on microbial metabolites, intestinal function, immune regulation, and host metabolism.

Time-restricted eating (TRE) has therefore emerged as a potential strategy for improving circadian alignment and metabolic regulation. By restricting food intake to a consistent daily window, TRE may strengthen the synchronization between feeding–fasting cycles and peripheral clocks and may modify microbial rhythmicity and metabolic activity. However, the contribution of the gut microbiota to these effects remains incompletely defined, and human studies have reported heterogeneous microbiota responses (Lotti et al., 2023) (**Table 5**).

**Table 5:** Chrononutritional interventions and their effects on gut microbiota and host physiology.

Intervention	Effects on gut microbiota	Proposed mechanisms	Commonly reported health outcomes	References
Time-restricted eating (TRE)	May influence microbial rhythmicity; May modify SCFA-producing taxa	Synchronization of peripheral clocks and feeding–fasting cycles	Improved insulin sensitivity and metabolic flexibility	(Matenchuk et al., 2020; Lotti et al., 2023)
Regular meal timing	May help preserve diurnal microbial oscillations	Stable nutrient availability and microbial substrate utilization	Potential effects on microbial diversity and immune homeostasis	(Lotti et al., 2023)
Avoidance of nighttime eating	Reduces circadian misalignment and microbial dysbiosis	Alignment between central and peripheral clocks	Lower inflammation and improved glucose metabolism	(Matenchuk et al., 2020; Lotti et al., 2023; Bajaj and Sharma, 2025)
Early time-restricted feeding	May be associated with changes in microbial diversity	Reinforcement of circadian gene expression	Improved cardiometabolic health	(Manoogian et al., 2022; Lotti et al., 2023)
Personalized chrononutrition	Potential optimization of microbiota according to chronotype and metabolic profile	Precision alignment of diet, microbiota, and circadian biology	Potential reduction in chronic disease risk	(Lotti et al., 2023; Jasar et al., 2024)

Microbial metabolites may represent one mechanism linking meal timing with host physiology. Changes in the timing of SCFA, secondary bile acid, and tryptophan-derived metabolite production may influence epithelial function, immune signaling, and metabolic pathways that interact with peripheral circadian clocks (Lotti et al., 2023; Bajaj and Sharma, 2025). Nevertheless, interpret these mechanisms cautiously because much of the mechanistic evidence comes from experimental models, while controlled long-term human studies remain limited.

The potential benefits of chrononutrition may also depend on individual characteristics. Chronotype, habitual diet, age, metabolic status, sleep patterns, medication use, and baseline gut microbiota composition may influence responses to changes in meal timing. Consequently, chrononutrition may ultimately require integration with individual dietary, metabolic, behavioral, and microbial characteristics rather than a universal feeding schedule (Lotti et al., 2023; Jasar et al., 2024).

### 4.4 Aging and medication exposure

#### 4.4.1 Aging and the gut microbiota

The gut microbiota undergoes continuous remodeling throughout the lifespan and contributes to host metabolism, intestinal barrier maintenance, immune regulation, and communication with extraintestinal organs. These changes are influenced not only by chronological age but also by diet, physical activity, medication exposure, comorbidities, living environment, and host physiological status. Consequently, aging-associated microbial patterns are highly heterogeneous and should not be interpreted as a uniform or inevitable transition toward dysbiosis (Donati Zeppa et al., 2023; Gyriki et al., 2025; Kanimozhi and Sukumar, 2025).

During aging, microbial composition changes may be accompanied by shifts in metabolic capacity, including pathways involved in carbohydrate fermentation, short-chain fatty acid (SCFA) production, amino acid metabolism, and bile acid transformation. These functional changes may influence intestinal barrier integrity, immune homeostasis, and systemic metabolism. However, taxonomic composition alone may not adequately capture the biological consequences of aging, as microbial functional capacity and resilience may be more closely related to health status than the presence or absence of individual bacterial taxa (Ling et al., 2022; Donati Zeppa et al., 2023; Gyriki et al., 2025).

##### 4.4.1.1 Lifespan trajectory and age-associated microbial changes

The gut microbiota develops and changes dynamically from early life through adulthood and older age. Early colonization is strongly influenced by delivery mode, infant feeding, host immune maturation, environmental exposure, and early dietary patterns, with *Bifidobacterium* frequently representing an important component of the infant microbiota. During adulthood, the microbial community becomes more diverse and functionally complex, whereas later life is often characterized by greater variability in microbial composition and reduced ecological stability (Gyriki et al., 2025; Kanimozhi and Sukumar, 2025).

In older adults, microbial composition changes often include shifts in the abundance of taxa involved in carbohydrate fermentation, SCFA production, and intestinal homeostasis, along with changes in facultative anaerobic and potentially inflammation-associated microorganisms. Some studies report reductions in SCFA-associated taxa, including members of Faecalibacterium, Roseburia, and Eubacterium, in association with aging, although these patterns are not consistently observed across populations. Similarly, changes in Bifidobacterium, Lactobacillus, and members of the Proteobacteria and Enterobacteriaceae have been described, but their direction and magnitude vary by health status, diet, medication exposure, and environmental conditions (Ling et al., 2022; Donati Zeppa et al., 2023; Gyriki et al., 2025).

Age-related changes in the gut microbiota are therefore better understood as alterations in microbial ecology and function rather than as a universal loss of specific “beneficial” bacteria. In particular, microbial diversity may remain relatively preserved in some healthy older adults, whereas marked alterations may occur in individuals with frailty, multimorbidity, institutionalization, or polypharmacy. These observations indicate that chronological age alone does not fully explain microbial variation in later life (Donati Zeppa et al., 2023; Gyriki et al., 2025).

Functional alterations may provide additional information about the biological consequences of microbial aging. Metagenomic studies suggest that pathways related to carbohydrate utilization, amino acid biosynthesis, SCFA production, and secondary bile acid metabolism may distinguish different health trajectories in older adults. In this context, preserving microbial metabolic capacity may help maintain physiological resilience even when substantial taxonomic differences are not apparent (Ling et al., 2022; Deng et al., 2025).

Recent evidence from long-lived adults further supports the importance of microbial functionality. Deng et al. (2025) reported enrichment of microbial pathways related to branched-chain amino acid biosynthesis in adults with preserved physical function, together with greater abundance of taxa including *Roseburia hominis*, *Eubacterium rectale*, and *Eubacterium eligens*. These findings suggest that preserved microbial metabolic functions may be associated with physical function and intrinsic capacity in advanced age. However, causal relationships between these microbial features and healthy aging remain to be established (Deng et al., 2025).

**Table 6** summarizes the age-associated microbial trajectory, highlighting commonly reported microbial patterns across life stages and their potential functional and health-related implications. Importantly, these patterns represent general observations rather than universal microbial signatures of aging, as substantial interindividual variability emerges, particularly in later life (Donati Zeppa et al., 2023; Gyriki et al., 2025).

**Table 6:** Age-associated gut microbiota patterns and commonly reported functional associations.

Life stage	Commonly reported patterns	Commonly reported functional/health associations	References
Early life	Early gut colonization is primarily by Bifidobacteria.	Supports digestion and immune development.	(Kanimozhi and Sukumar, 2025)
Adulthood	Microbial diversity increases; dominant genera/phyla include Firmicutes, Bacteroidetes, Actinobacteria, and Proteobacteria.	Establishment of a mature, adult-like community, with relatively stable microbial composition and function compared with early-life stages.	(Gyriki et al., 2025; Kanimozhi and Sukumar, 2025)
Older age (general trend)	Altered microbial diversity and community stability; shifts in taxa involved in carbohydrate fermentation, SCFA production, and intestinal homeostasis, with substantial interindividual variability.	Some older populations show altered gut barrier integrity and increased intestinal permeability, along with markers of systemic inflammation (“inflammaging”). Reductions in SCFA-associated microbial functions have also been reported, although their relationship with inflammation and neurodegenerative outcomes remains uncertain.	(Gyriki et al., 2025; Kanimozhi and Sukumar, 2025; Donati Zeppa et al., 2023)
Frailty/morbidity context	Dysbiosis in older age is associated with increased intestinal permeability and immune activation; older adults exhibit greater interindividual variability.	Associations with chronic inflammation, frailty, multimorbidity, and altered intestinal permeability have been reported, with substantial interindividual variability.	(Donati Zeppa et al., 2023; Gyriki et al., 2025)
Centenarians/long-lived adults	Centenarians/oldest-old often show distinctive profiles (e.g., *Prevotella* enterotype predominance reported in one cohort; enrichment of taxa such as *Akkermansia* reported); long-lived adults (90–98) reported enrichment in *Akkermansia* and *Bifidobacterium*.	Associations with chronic inflammation, frailty, multimorbidity, and altered intestinal permeability have been reported, with substantial interindividual variability.	(Donati Zeppa et al., 2023; Deng et al., 2025; Gyriki et al., 2025)

##### 4.4.1.2 Gut microbiota, inflammaging, and healthy aging

One of the most relevant consequences of age-associated changes in the gut microbiota is their potential interaction with chronic low-grade inflammation, commonly called inflammaging. Aging is accompanied by progressive changes in immune regulation, and alterations in intestinal microbial communities may contribute through changes in epithelial barrier function, microbial metabolites, and exposure to microbial-associated molecular patterns (Ling et al., 2022; Donati Zeppa et al., 2023).

Reduced abundance or activity of microorganisms involved in SCFA production may affect epithelial integrity and immune regulation, particularly by altering butyrate availability. Butyrate supports epithelial energy metabolism, tight-junction regulation, and immune homeostasis, while microbial alterations that reduce SCFA production may alter the intestinal environment and host inflammatory responses. However, the relationship between reduced SCFA production and aging-associated inflammation is not necessarily linear and may depend on dietary substrate availability and the resident microbiota’s functional capacity (Donati Zeppa et al., 2023; Gyriki et al., 2025).

Age-associated alterations in intestinal barrier function may further influence systemic immune signaling. Increased intestinal permeability can facilitate greater exposure to microbial products such as lipopolysaccharide (LPS), which may activate pattern-recognition receptors, including Toll-like receptor 4 (TLR4), and contribute to inflammatory signaling. These mechanisms provide a plausible link between intestinal microbial alterations and systemic inflammation, although human evidence has not established a single causal pathway linking microbiota changes to inflammaging (Ling et al., 2022; Donati Zeppa et al., 2023).

The potential consequences of these interactions extend beyond the gastrointestinal tract. Age-associated microbial alterations and chronic inflammatory processes have been associated with conditions including frailty, sarcopenia, metabolic dysfunction, cardiovascular disease, neurodegenerative disorders, and impaired immune responses. Nevertheless, these associations are influenced by multiple factors, including comorbidities, nutritional status, physical activity, medication use, and socioeconomic and environmental conditions, making it difficult to attribute these outcomes to the microbiota alone (Donati Zeppa et al., 2023; Gyriki et al., 2025).

Healthy aging provides an important counterpoint to the concept of inevitable age-related dysbiosis. Studies of centenarians and other exceptionally long-lived adults have identified microbial profiles that differ from those observed in some younger or older populations, including enrichment of taxa such as *Akkermansia* and *Bifidobacterium* and preservation of microbial pathways associated with SCFA and secondary bile acid metabolism. However, these characteristics are not consistent across geographical populations, suggesting that longevity-associated microbiota features may reflect interactions among microbial ecology, diet, environment, genetics, medication exposure, and health status rather than a universal microbial signature of longevity (Donati Zeppa et al., 2023; Deng et al., 2025; Gyriki et al., 2025).

For example, adults aged 90–98 years with preserved physical function have been reported to maintain microbial characteristics distinct from those observed in younger-old populations, including enrichment of *Akkermansia* and *Bifidobacterium* and associations between microbial features and physical or intrinsic capacity. These findings suggest that preserving microbial function and ecological resilience may matter more for healthy aging than the abundance of any single taxon (Deng et al., 2025).

Importantly, proposed microbial signatures of longevity vary among populations. Differences in diet, geography, lifestyle, medication use, health status, and analytical approaches may contribute to distinct microbial configurations among long-lived individuals. Therefore, microbial characteristics associated with exceptional longevity should be considered population- and context-dependent rather than universal determinants of lifespan (Donati Zeppa et al., 2023; Gyriki et al., 2025).

Current evidence suggests that aging is associated with substantial remodeling of gut microbial ecology and function, but these changes are highly heterogeneous. Rather than defining aging by a uniform loss of microbial diversity or depletion of specific “beneficial” microorganisms, a more informative framework considers microbial functional capacity, metabolic resilience, intestinal barrier integrity, and interactions with immune and metabolic pathways. This perspective also highlights the importance of host and environmental context and provides a rationale for investigating individualized strategies to preserve microbial and physiological resilience during aging (Donati Zeppa et al., 2023; Deng et al., 2025; Gyriki et al., 2025).

#### 4.4.2 Medication exposure: antibiotics

Medications are important external modifiers of the gut microbiota. Antibiotics are among the most potent and extensively studied examples, although non-antibiotic drugs can also alter microbial composition and function (Gommers et al., 2019). Because antibiotics also affect commensal microorganisms, their use can alter the abundance, composition, and functional activity of the gut microbial community. The resulting perturbation may affect microbial metabolic pathways, colonization resistance, and host–microbiota interactions, with the magnitude and persistence of these effects depending on the antibiotic agent, dose, treatment duration, and characteristics of the host and baseline microbiota (Dahiya and Nigam, 2023; Lathakumari et al., 2024; Cusumano et al., 2025).

Antibiotic-induced dysbiosis can involve reduced microbial diversity and shifts in the relative abundance of multiple bacterial groups. However, the direction and magnitude of these changes vary substantially by individual and antibiotic class. Broad-spectrum agents and repeated or prolonged exposure generally produce more extensive ecological perturbations than short courses of narrower-spectrum antibiotics. Importantly, microbial recovery after treatment is often incomplete or heterogeneous, and some alterations may persist beyond antimicrobial exposure (Mulder et al., 2020; Lathakumari et al., 2024; Cusumano et al., 2025). The main determinants and consequences of antibiotic-induced gut dysbiosis are summarized in **Table 7**.

**Table 7:** Determinants and consequences of antibiotic-induced gut dysbiosis.

Determinant	Reported effect on the gut microbiota	Functional consequences	Reported clinical implications	References
Broad-spectrum antibiotics	Greater loss of microbial diversity and depletion of commensal bacteria	Reduced ecosystem resilience	Increased risk of persistent dysbiosis	(Lathakumari et al., 2024; Cusumano et al., 2025)
Prolonged or repeated antibiotic exposure	Delayed microbial recovery and long-term community restructuring	Persistent metabolic alterations	Higher susceptibility to recurrent dysbiosis	(Mulder et al., 2020)
Antibiotic class	Class-specific depletion of selected commensal taxa and expansion of opportunistic microorganisms	Altered SCFA production and microbial metabolism	Variable ecological recovery	(Mulder et al., 2020; Huang et al., 2024)
Host-related factors (age, diet, baseline microbiota, comorbidities)	Variable susceptibility to microbial disruption	Differences in resilience and recovery	Interindividual variability in dysbiosis severity	(Lathakumari et al., 2024; Cusumano et al., 2025)
Reduced beneficial bacteria (*Bifidobacterium*, *Lactobacillus*, *Faecalibacterium*)	Loss of colonization resistance and SCFA production	Impaired barrier integrity and immune regulation	Increased risk of antibiotic-associated complications	(Dahiya and Nigam, 2023; Cusumano et al., 2025)

The functional consequences of antibiotic exposure may be more relevant than taxonomic changes alone. Antibiotic-associated perturbations can affect microbial pathways involved in SCFA production, bile acid metabolism, vitamin biosynthesis, and other host–microbiota metabolic interactions. Loss of microbial functional capacity may also weaken colonization resistance and alter the intestinal environment, potentially increasing susceptibility to opportunistic microorganisms and antibiotic-associated complications (Dahiya and Nigam, 2023; Lathakumari et al., 2024).

The clinical consequences of antibiotic-induced microbiota disruption depend strongly on the host context. Age, nutritional status, comorbidities, immune function, previous antibiotic exposure, diet, and baseline microbiota composition may influence both susceptibility to disruption and subsequent recovery. Thus, individuals receiving the same antibiotic regimen may experience markedly different degrees and durations of microbiota perturbation, reinforcing the importance of interindividual variability in antibiotic–microbiota interactions (Lathakumari et al., 2024; Cusumano et al., 2025).

Antibiotic exposure can also create ecological conditions that favor the persistence or expansion of antimicrobial-resistant microorganisms. Depletion of susceptible members of the microbial community may reduce ecological competition and create opportunities for resistant organisms to proliferate. At the same time, antibiotic exposure can select for or enrich antibiotic resistance genes within the gut microbial community, extending treatment consequences beyond temporary changes in microbial composition (Lamberte and van Schaik, 2022; Dongre et al., 2025).

Recovery of the gut microbiota after antibiotic treatment is therefore an important component of microbiome resilience. Although microbial communities can partially recover after treatment cessation, recovery trajectories differ by antibiotic class, treatment history, host characteristics, and baseline ecological structure. Some studies have reported persistent alterations in diversity or community composition months or even years after antibiotic exposure, emphasizing that antibiotic-induced dysbiosis should not necessarily be viewed as a transient disturbance (Mulder et al., 2020; Cusumano et al., 2025).

##### 4.4.2.1 Antibiotic resistance, the gut resistome, and strategies for microbiota restoration

Antibiotic-induced microbiota disruption extends beyond the loss of susceptible microorganisms by influencing the gut resistome, defined as the collection of antimicrobial resistance genes (ARGs) present within the microbial community. The intestinal microbiota can act as a reservoir of ARGs, including genes carried by commensal microorganisms that may potentially be transferred to opportunistic or pathogenic bacteria. Antibiotic exposure can exert selective pressure that favors the persistence or enrichment of resistant populations and resistance determinants (Lamberte and van Schaik, 2022; Dongre et al., 2025).

The relationship between antibiotic exposure and antimicrobial resistance is complex because resistance can persist even when the original antibiotic is no longer present. Selection pressure, horizontal gene transfer, ecological restructuring, and persistence of resistant microbial populations may contribute to prolonged changes in the gut resistome. Consequently, evaluate the effects of antibiotic treatment not only on immediate microbiota composition but also on changes in the abundance and diversity of ARGs (Lamberte and van Schaik, 2022; Dongre et al., 2025).

Strategies to restore the gut microbiota after antibiotic treatment have therefore attracted considerable interest. Clinical and experimental studies have investigated dietary interventions, prebiotics, probiotics, synbiotics, postbiotics, and microbiota-based therapies. However, their efficacy is inconsistent, and restoring microbial diversity does not necessarily mean complete recovery of microbial function or the resistome (Dahiya and Nigam, 2023; Lathakumari et al., 2024).

The use of probiotics after antibiotic treatment illustrates this complexity. Probiotic supplementation may support the recovery of selected microbial populations or reduce the risk of specific antibiotic-associated complications in some clinical settings. Still, effects are highly strain-specific and dependent on the host and clinical context. Moreover, probiotic administration does not necessarily restore the individual’s original microbiota or eliminate antibiotic-associated changes in the resistome (Dahiya and Nigam, 2023).

Dietary strategies may provide an additional approach to supporting microbial recovery. Adequate intake of fermentable dietary substrates can provide ecological resources for the expansion and metabolic activity of microorganisms that utilize complex carbohydrates and produce SCFAs. Nevertheless, dietary interventions should be considered in relation to the individual’s tolerance, clinical condition, baseline microbiota, and antibiotic exposure history rather than as universal microbiota-restoration strategies (Dahiya and Nigam, 2023; Lathakumari et al., 2024).

Researchers are also investigating more targeted microbiota-based approaches, particularly for severe or recurrent antibiotic-associated dysbiosis. These include fecal microbiota transplantation and defined microbial consortia designed to restore colonization resistance and microbial functionality. Although such approaches have demonstrated clinical utility in selected conditions, their application should be distinguished from generalized microbiota “restoration,” because successful recovery depends on the clinical indication, microbial donor or consortium characteristics, host factors, and the specific ecological functions being targeted (Lathakumari et al., 2024).

A key challenge is defining what constitutes successful microbiota recovery. Restoration of taxonomic diversity alone may not adequately reflect recovery of microbial metabolic functions, ecological resilience, or the resistome. Future studies should therefore integrate taxonomic, functional, metabolomic, and antimicrobial-resistance measurements to evaluate recovery more comprehensively and identify individuals at increased risk of persistent antibiotic-associated alterations (Lamberte and van Schaik, 2022; Dongre et al., 2025).

## 5 The challenge of personalization: from general advice to targeted intervention

### 5.1 Interindividual variability

Interindividual variability has emerged as a major challenge in microbiome research and precision nutrition. Although dietary and lifestyle interventions are commonly recommended to improve health, individuals may respond markedly differently to the same intervention. These heterogeneous outcomes challenge the traditional “one-size-fits-all” approach to nutrition and highlight the need to understand the biological factors that contribute to individual responsiveness. Current evidence indicates that this variability results from the complex interaction among host genetics, age, sex, health status, habitual diet, lifestyle, environmental exposures, medication use, and, importantly, the composition and functional capacity of the gut microbiota (Hernández-Calderón et al., 2022; Bianchetti et al., 2023).

The gut microbiota may contribute substantially to differences in dietary responses because microorganisms digest and biotransform dietary components into biologically active metabolites. Individuals differ not only in microbial taxonomic composition but also in microbial gene content, metabolic pathways, and capacity to transform specific dietary substrates. Consequently, the same food or dietary component may generate different microbial metabolite profiles and, potentially, different physiological responses among individuals. Such variability has been reported for outcomes including body weight, glycemic regulation, inflammation, and cardiometabolic health, although the relative contribution of the microbiota compared with host and environmental factors remains incompletely defined (Hernández-Calderón et al., 2022; Bianchetti et al., 2023).

A particularly relevant example is the interindividual variability in the metabolism of dietary polyphenols. Gut microorganisms can transform polyphenols into metabolites that may differ substantially from their parent compounds in terms of bioavailability and biological activity. However, the capacity to produce specific microbial-derived metabolites varies considerably among individuals. For example, only a subset of individuals converts soy isoflavones into equol. Similarly, the production of urolithins from ellagitannins depends on the presence and activity of specific microbial communities. Similar variability has been described for other bioactive compounds, including curcumin, illustrating how differences in microbial metabolic capacity may contribute to heterogeneous responses to dietary bioactives (Morand, 2024).

These observations have contributed to the concept of metabotypes, which classify individuals by their capacity to generate specific microbial-derived metabolites rather than solely by taxonomic composition. Importantly, metabotypes should not necessarily be interpreted as discrete and immutable categories. Individuals may produce multiple metabolites within a given metabolic pathway but in different proportions, reflecting variation in microbial functionality and host metabolism. Consequently, comprehensive metabolomic profiling may provide a more nuanced characterization of individual metabolic phenotypes than simple producer/non-producer classifications (Morand, 2024).

An additional challenge is that microbial composition alone may not reliably predict physiological responses. Taxonomically distinct microbial communities can exhibit overlapping metabolic functions because different microorganisms may encode similar biochemical pathways, a phenomenon commonly referred to as functional redundancy. Conversely, individuals with relatively similar taxonomic profiles may differ in microbial gene expression, metabolic activity, or metabolite production. Therefore, functional characteristics and metabolic outputs may provide complementary information to taxonomic composition when evaluating host–microbiota interactions and dietary responsiveness (Bianchetti et al., 2023).

Collectively, these findings indicate that individual responses to dietary interventions emerge from interactions among multiple biological and environmental determinants rather than from a single microbial or host characteristic (**Figure 5**). Differences in microbial composition, functional capacity, substrate utilization, and metabolite production occur within a broader context that includes host physiology, habitual diet, lifestyle, medication exposure, and environmental factors. Thus, understanding interindividual variability requires moving beyond identifying individual taxa toward an integrated assessment of microbial functions and host responses (Bianchetti et al., 2023; Morand, 2024).

**Figure 5:**
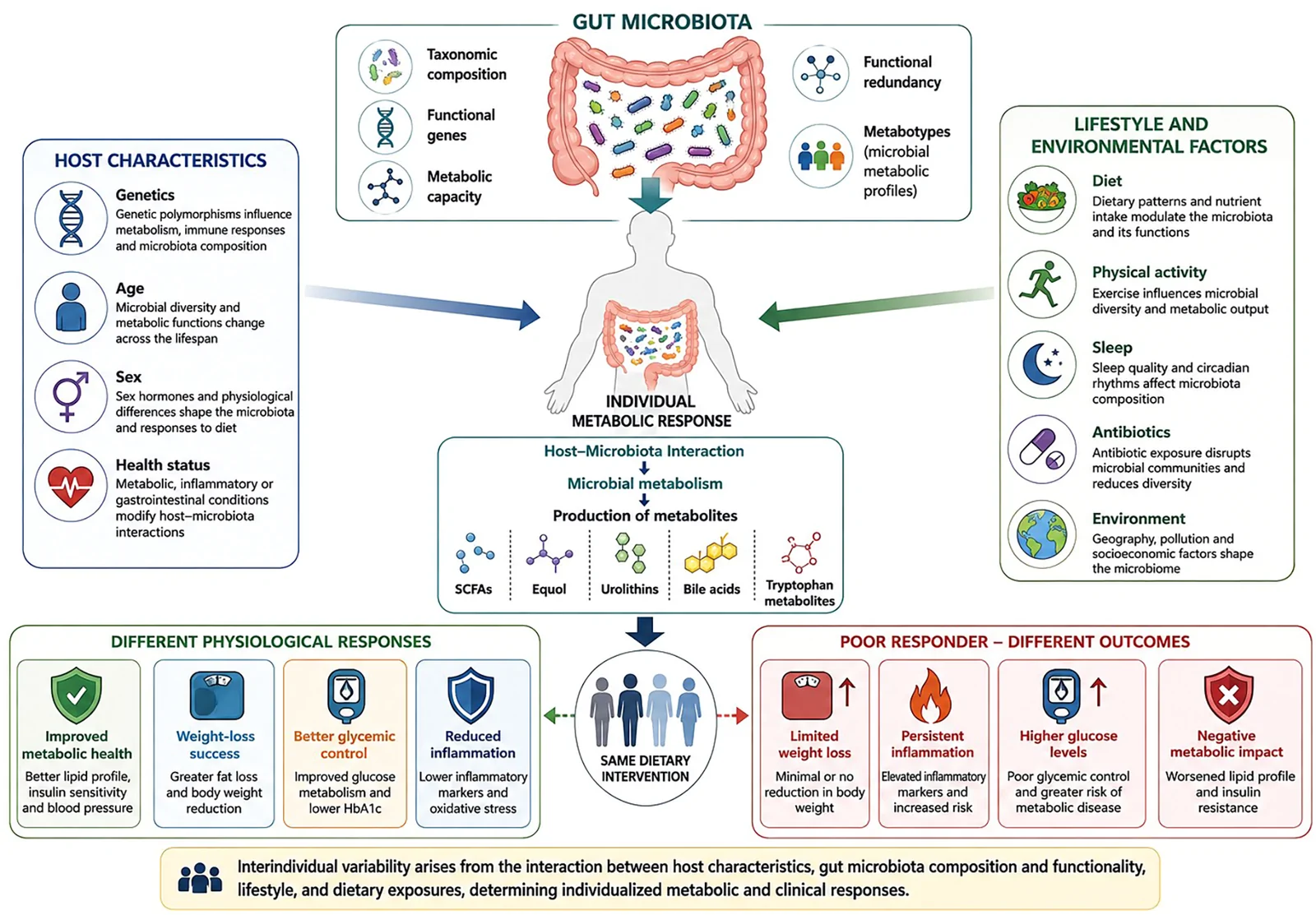
Biological determinants of interindividual variability in microbiota-mediated dietary responses.

These considerations also have important implications for nutritional research. Much of the available evidence on interindividual variability comes from *post hoc* analyses of clinical trials, while relatively few studies have been designed to characterize and predict differential responses. Improved baseline characterization, participant stratification, metabotyping, longitudinal sampling, and integration of multi-omics data may help identify biological determinants of responsiveness and improve the interpretation of heterogeneous intervention outcomes (Morand, 2024).

### 5.2 Personalized nutrition

Recognizing substantial interindividual variability has stimulated growing interest in personalized nutrition, an approach that adapts dietary recommendations to individual biological, metabolic, behavioral, and environmental characteristics. Unlike conventional population-based dietary recommendations, personalized nutrition aims to account for differences in nutritional requirements and responses among individuals. Within this framework, the gut microbiota represents one potentially informative component because its metabolic activity can influence the transformation of dietary substrates and the generation of bioactive metabolites (Nogal et al., 2021; Livingstone et al., 2022).

Microbiome-informed nutrition therefore requires consideration of both microbial composition and functionality. Taxonomic profiling describes the microorganisms present in an individual, whereas metagenomic, transcriptomic, and metabolomic approaches provide complementary information on microbial functional potential, activity, and metabolite production. Integrating these datasets with host characteristics, dietary intake, metabolic phenotype, and clinical variables may improve the identification of response patterns and help distinguish individuals more or less likely to benefit from a given dietary intervention (Livingstone et al., 2022).

An important application of this approach is identifying responders and non-responders to specific dietary components. Differences in microbial metabolic capacity may influence the bioavailability and biological effects of food-derived compounds, particularly polyphenols and other bioactive molecules. Consequently, metabotyping may provide a framework for stratifying individuals according to their capacity to generate specific microbial metabolites and for evaluating whether these metabolic profiles are associated with differential health responses (Narduzzi et al., 2022; Morand, 2024).

Predictive models represent another emerging strategy. Machine learning and other data-driven approaches can integrate high-dimensional datasets involving microbiota composition, microbial genes, metabolites, dietary information, and host characteristics to identify patterns associated with individual responses. These approaches can improve prediction of dietary outcomes and identify subgroups with distinct response profiles. However, researchers must evaluate predictive performance in independent populations, and models developed from highly controlled cohorts may not generalize to broader or clinically diverse populations (Nogal et al., 2021; Livingstone et al., 2022).

Nevertheless, translating microbiome-informed precision nutrition into clinical practice faces important challenges. First, the gut microbiota is dynamic and can change according to diet, lifestyle, medication exposure, illness, and other environmental factors. Second, methodological differences in sample collection, sequencing, bioinformatic analysis, dietary assessment, and metabolite measurement can complicate comparisons among studies. Third, associations between microbial features and health outcomes do not necessarily establish causality. Finally, identifying a microbial signature associated with a dietary response does not necessarily show that modifying that microbial feature will reproduce the predicted clinical benefit (Nogal et al., 2021; Livingstone et al., 2022).

Another important limitation is that researchers often assess interindividual variability retrospectively rather than incorporating it prospectively into study design. Stratified randomization, crossover designs, longitudinal interventions, N-of-1 trials, and adaptive trial designs may provide complementary approaches for characterizing individual responses and identifying factors associated with differential efficacy. In particular, N-of-1 approaches can directly evaluate within-person responses to dietary interventions, whereas adaptive and stratified designs may help identify response subgroups in larger populations (Morand, 2024).

Accordingly, personalized nutrition should currently be considered an emerging framework rather than a fully established clinical paradigm. The available evidence supports the potential value of integrating gut microbiota data with host, dietary, metabolic, and lifestyle characteristics. Still, further prospective and longitudinal studies are required to determine which microbial and metabolic features can reproducibly predict clinically meaningful responses. Standardizing analytical methods, externally validating predictive models, and demonstrating improved clinical outcomes will be essential before microbiome-informed dietary recommendations can be broadly implemented (Nogal et al., 2021; Livingstone et al., 2022).

Ultimately, microbiome-informed nutrition should not aim to define a single “optimal” microbiota or prescribe diets solely based on microbial composition. Rather, the goal should be to identify reproducible host–microbiota functional signatures that can help predict and modulate individual responses to dietary interventions. This perspective shifts personalized nutrition from a taxonomy-centered approach to one focused on microbial functionality, metabolic capacity, and host response, providing a more biologically meaningful framework for developing targeted nutritional strategies.

## 6 Conclusions

The evidence reviewed in this article supports viewing the gut microbiota as a dynamic component of the host environment that responds continuously to dietary exposures, lifestyle behaviors, environmental factors, medication use, and physiological changes across the lifespan. These factors interact to influence microbial community structure, functional capacity, metabolic activity, intestinal homeostasis, and host–microbiota communication. Therefore, the effects of dietary and lifestyle exposures should be interpreted within the broader host–microbiota context rather than as isolated factors.

A central concept emerging from the evidence is that microbiota responses are highly context-dependent and heterogeneous among individuals. Differences in host characteristics, habitual diet, lifestyle, medication history, baseline microbiota, and microbial metabolic capacity may contribute to distinct responses to similar interventions. Moreover, taxonomic composition alone may not capture these differences, underscoring the importance of considering microbial functionality and host phenotype when evaluating microbiota-mediated effects.

Future research should move beyond descriptive associations toward a more mechanistic understanding of host–microbiota interactions. Longitudinal human studies, standardized methodologies, repeated sampling, and integration of microbiome, metabolomic, dietary, physiological, and clinical data will be essential to identify reproducible determinants of individual responses. Prospective validation of predictive models and demonstration of clinically meaningful benefits will also be necessary to translate microbiome-informed interventions into practice.

In this context, personalized nutrition represents a promising but evolving framework for developing targeted nutritional strategies. Its future application should focus not on identifying a single “optimal” microbiota, but on determining microbial functions, metabolic signatures, and host characteristics that can reliably predict and modulate individual responses. Ultimately, understanding these functional interactions may support more precise interventions aimed at maintaining microbial homeostasis, reducing disease risk, promoting healthy aging, and improving health across diverse populations.
